# Classification of pre-metabolic syndrome and metabolic syndrome using machine learning and routine clinical variables in Thai adults

**DOI:** 10.1371/journal.pone.0359283

**Published:** 2026-09-28

**Authors:** Metha Yaikwawong, Khanittha Kamdee, Somlak Chuengsamarn

**Affiliations:** 1 Department of Pharmacology, Faculty of Medicine Siriraj Hospital, Mahidol University, Bangkok, Thailand; 2 Division of Endocrinology and Metabolism, Faculty of Medicine, HRH Princess Maha Chakri Sirindhorn Medical Center, Srinakharinwirot University, Nakhon Nayok, Thailand; University of Diyala College of Medicine, IRAQ

## Abstract

**Background:**

Metabolic syndrome (MetS) and pre-metabolic syndrome (pre-MetS) are increasingly recognized as important stages of metabolic dysfunction in Asian populations. Conventional diagnostic approaches rely on fixed threshold criteria, whereas metabolic dysfunction occurs along a continuous spectrum. Machine learning (ML) can integrate multidimensional clinical data and model nonlinear relationships among clinical variables, potentially providing complementary information for distinguishing metabolic states. This study evaluated ten ML algorithms for distinguishing pre-MetS from MetS in Thai adults and identified features contributing most strongly to model-based classification.

**Methods:**

In this single-center retrospective cross-sectional study, 657 Thai adults were classified as pre-MetS (*n* = 359) or MetS (*n* = 298). Pre-MetS was operationally defined as the presence of at least one IDF-defined metabolic abnormality without fulfilling the complete International Diabetes Federation (IDF) criteria for MetS. Twenty-four clinical and biochemical features selected using the Boruta algorithm were evaluated across ten supervised ML algorithms. Models were trained using an 80% training dataset with repeated 10-fold cross-validation (10 repetitions) for hyperparameter tuning and subsequently evaluated using an independent 20% hold-out test dataset (*n* = 132). All preprocessing steps, including z-score normalization and feature selection, were performed exclusively within the training dataset to prevent data leakage.

**Results:**

Among the ten evaluated algorithms, ensemble methods achieved the highest classification performance. XGBoost demonstrated the highest discrimination (AUC = 0.986, 95% CI: 0.970–0.997), highest accuracy (0.932, 95% CI: 0.885–0.969), and lowest Brier score (0.050, 95% CI: 0.026–0.080), followed by the neural network (AUC = 0.948, 95% CI: 0.906–0.983) and random forest (AUC = 0.942, 95% CI: 0.905–0.974). SHAP analysis identified systolic blood pressure, waist circumference, fasting plasma glucose, sex, and TyG-WHtR as the features contributing most strongly to XGBoost classification. Sensitivity analyses demonstrated progressively reduced discrimination after excluding predictors that overlapped with MetS diagnostic components and related derived indices, although substantial discrimination was retained after removal of these overlapping features.

**Conclusions:**

Among the ten evaluated algorithms, classification performance varied substantially across models, with XGBoost achieving the highest performance and support vector machine models showing the lowest discrimination. These findings indicate that algorithm choice may influence classification performance in metabolic classification tasks. However, the observed performance should be interpreted cautiously because several influential predictors overlap with, or are derived from, the MetS diagnostic criteria, and the study lacked external validation. The findings are therefore exploratory and require validation in multicenter cohorts using predictor sets that minimize overlap with the diagnostic definition before clinical implementation can be considered.

## Introduction

Cardiometabolic diseases, including type 2 diabetes mellitus (T2DM) and metabolic syndrome (MetS), represent a major global health burden and contribute substantially to morbidity, mortality, and healthcare costs worldwide. MetS is characterized by a cluster of interrelated metabolic abnormalities, including central obesity, dyslipidemia, hypertension, and hyperglycemia, which increase the risk of cardiovascular disease and diabetic complications [1,2]. Globally, the prevalence of MetS has increased substantially during recent decades in parallel with rising rates of obesity, population aging, physical inactivity, and unhealthy dietary patterns [3,4]. The consequences extend beyond clinical complications, as MetS is associated with increased healthcare utilization, long-term pharmacological treatment, reduced productivity, diminished quality of life, and substantial economic burden on healthcare systems [5]. Consequently, early recognition of individuals with emerging metabolic dysfunction has become a public health priority aimed at reducing future cardiometabolic morbidity and mortality [3,6].

The burden of MetS is particularly pronounced in Asian populations, where rapid economic development, urbanization, and lifestyle transitions have accelerated the prevalence of obesity and diabetes-related disorders. In Southeast Asia, increasing consumption of energy-dense diets, reduced physical activity, and demographic aging have contributed to a growing cardiometabolic disease burden [7,8]. Thailand has experienced similar trends, with national health surveys demonstrating a substantial burden of metabolic abnormalities among adults. Data from the fourth Thai National Health Examination Survey (NHES IV) showed that MetS affected 23.2% of Thai adults aged ≥20 years, with prevalence estimates of 19.5% in men and 26.8% in women [9]. Similarly, a nationally representative study of Thai adults aged ≥35 years reported an age-standardized MetS prevalence of 24.0% according to IDF criteria, although prevalence increased to 32.6% when the NCEP-ATP III criteria were applied [10]. These differences highlight the influence of diagnostic definitions on estimates of the population burden of MetS. The associated healthcare expenditures, loss of productivity, and increased risk of cardiovascular disease and T2DM place considerable strain on individuals, families, and healthcare systems [11]. Therefore, improved strategies for identifying individuals at different stages of metabolic dysfunction are needed in the Thai context.

The metabolic abnormalities underlying MetS often accumulate over time rather than emerging simultaneously. Before fulfilling the complete diagnostic criteria for MetS, individuals may exhibit an intermediate metabolic state characterized by the presence of some, but not all, metabolic abnormalities. This transitional state may represent an early phase of metabolic dysfunction and a potential opportunity for preventive intervention [7].

This intermediate condition has been described as pre-metabolic syndrome (pre-MetS). Although pre-MetS has gained increasing attention as a potentially important stage in the trajectory toward overt metabolic disease, it is not as universally standardized as MetS, and definitions vary among studies [8,9]. Consequently, a clear operational definition is required for research purposes to facilitate consistent classification and interpretation of findings. In the present study, pre-MetS was operationally defined as the presence of at least one IDF-defined metabolic abnormality without fulfilling the complete IDF criteria for MetS. Central obesity was not required for classification as pre-MetS [8,10].

The clinical significance of pre-MetS lies in its potential to represent an early stage of metabolic deterioration rather than a metabolically healthy state. Individuals with pre-MetS may already demonstrate evidence of insulin resistance, central adiposity, dyslipidemia, or impaired glucose regulation and may be at elevated risk of progression to MetS, T2DM, and cardiovascular disease [8]. Therefore, pre-MetS was selected as the comparison group because it represents a clinically relevant intermediate stage along the continuum of metabolic dysfunction. Distinguishing individuals with pre-MetS from those with established MetS may provide insight into differences in metabolic status and identify opportunities for preventive intervention before further metabolic deterioration occurs [9].

Conventional diagnosis of MetS relies on established threshold-based criteria such as those proposed by the International Diabetes Federation (IDF), which classify individuals according to predefined cut-off values for waist circumference, blood pressure, plasma glucose, triglycerides, and HDL-C [11]. These criteria remain an established clinical framework and provide a simple and practical approach to MetS classification. However, metabolic dysfunction exists on a biological continuum, whereas threshold-based definitions categorize individuals into discrete groups. As a result, individuals with similar underlying metabolic profiles may be classified differently depending on whether specific diagnostic cut-offs are exceeded [12]. Furthermore, conventional threshold-based criteria do not quantify the relative contribution or potential interactions among metabolic variables and may not fully represent the multidimensional nature of metabolic dysfunction [13,14]. Therefore, although established diagnostic criteria remain the clinical standard for MetS classification, complementary analytical approaches may provide additional insight into how multiple routinely collected variables collectively characterize different stages of metabolic dysfunction.

Machine learning (ML) provides a complementary analytical approach that can integrate numerous demographic, anthropometric, and biochemical variables simultaneously while identifying complex nonlinear relationships and interactions among features. Importantly, the purpose of ML in the present study was not to replace established IDF diagnostic criteria. Rather, ML was evaluated as a tool for classification and characterization of existing metabolic states, allowing assessment of how routinely collected clinical variables collectively distinguish individuals with pre-MetS from those with MetS. Consistent with the cross-sectional study design, the developed models were intended to classify existing metabolic status rather than predict future disease progression or cardiometabolic outcomes.

In addition, ML models can provide information regarding feature importance, thereby identifying variables that contribute most strongly to model-based metabolic classification. Such information may improve understanding of the metabolic patterns associated with more advanced metabolic states and support the development of simplified screening strategies using routinely available clinical data [15–17]**.** Among the variables increasingly investigated in cardiometabolic research are composite indices that combine anthropometric and biochemical parameters. The triglyceride-glucose (TyG) index and its derivatives, including TyG-waist circumference (TyG-WC) and TyG-waist-to-height ratio (TyG-WHtR), have emerged as practical surrogate markers of insulin resistance and visceral adiposity and have demonstrated associations with MetS, T2DM, and cardiovascular risk [18–20]. These indices are particularly attractive because they can be calculated using inexpensive and routinely available clinical measurements. However, several composite indices incorporate variables that overlap with diagnostic components of MetS, particularly waist circumference, triglycerides, and fasting plasma glucose [11,18–20]. Consequently, apparent improvements in classification performance may partly reflect overlap with the reference definition rather than entirely independent discriminatory information. Classification performance should therefore be interpreted cautiously, and sensitivity analyses are necessary to evaluate the extent to which such overlap influences model discrimination [21].

Unlike previous studies that have primarily focused on distinguishing MetS from healthy individuals, the present study specifically evaluated classification between pre-MetS and MetS, representing a more clinically challenging distinction along the spectrum of metabolic dysfunction. Furthermore, we systematically compared ten machine-learning algorithms and conducted sensitivity analyses to assess the influence of diagnostic overlap on classification performance. These methodological approaches provide additional insight into the potential utility and limitations of machine learning for metabolic classification in Thai adults.

Despite growing interest in ML applications for metabolic disease assessment, relatively limited evidence is available from Southeast Asian populations, particularly Thailand. Population-specific investigation is important because Asian individuals may develop insulin resistance, visceral adiposity, and cardiometabolic complications at lower body mass index levels than Western populations. These differences in body composition and metabolic characteristics suggest that findings derived from Western cohorts may not be directly generalizable to Thai adults [22,23]. Furthermore, evidence regarding ML-based classification specifically between pre-MetS and MetS remains limited.

Therefore, this study aimed to: (1) compare the clinical and metabolic characteristics of Thai adults with pre-MetS and MetS; (2) develop and evaluate multiple machine-learning models for classification of these existing metabolic states using routinely collected clinical variables; and (3) identify the features contributing most strongly to model-based classification of metabolic status.

## Methods

### Study design and ethical approval

This single-center retrospective cross-sectional study analyzed routinely collected clinical data from individuals attending the outpatient clinic at HRH Princess Maha Chakri Sirindhorn Medical Center, Srinakharinwirot University, Nakhon Nayok, Thailand, between January 2021 and December 2022. The study was approved by the Institutional Review Board of Srinakharinwirot University (SWUEC-683072) and was conducted in accordance with the Declaration of Helsinki.

Written informed consent permitting the use of clinical data for future research was obtained from all participants at the time of routine clinical enrollment. For the present study, de-identified clinical data were retrospectively extracted from the hospital database on 20 September 2025. Because only anonymized data were analyzed and no participant contact occurred, the Institutional Review Board waived the requirement for additional informed consent for this retrospective analysis.

### Participants

A total of 720 consecutive adults attending the outpatient clinic between January 2021 and December 2022 were screened for eligibility**.** Consecutive sampling was used, whereby all potentially eligible individuals attending the clinic during the study period were considered for inclusion rather than selecting participants according to predetermined sampling quotas.

All participants were recruited from a single tertiary-care academic medical center. Demographic, clinical, anthropometric, and biochemical data were retrospectively obtained from the institutional electronic medical record system.

Eligible participants were aged ≥18 years, classified as having pre-metabolic syndrome (pre-MetS) or metabolic syndrome (MetS), and had complete demographic, anthropometric, clinical, and biochemical data required for analysis. Individuals younger than 18 years were excluded because diagnostic criteria for MetS, waist circumference thresholds, and cardiometabolic risk profiles differ substantially between pediatric and adult populations. Restricting the study to adults ensured consistent application of the International Diabetes Federation (IDF) criteria and reduced population heterogeneity.

Participants were excluded if they were pregnant or lactating; had an acute illness within the preceding month; had chronic inflammatory disease, active infection, malignancy, severe hepatic or renal dysfunction, or endocrine disorders known to affect metabolic status; or had incomplete clinical or biochemical data. These exclusion criteria were identified from electronic medical records, physician diagnoses, laboratory test results, and clinician documentation available in the hospital information system.

Of the 720 individuals screened, 63 were excluded because of incomplete biochemical data (*n* = 41), incomplete anthropometric measurements (*n* = 22). Consequently, 657 participants were included in the final analysis, comprising 359 individuals with pre-MetS and 298 with MetS. The participant selection process is illustrated in S1 Fig**.**

Because this study analyzed an existing retrospective clinical database, no formal a priori sample size calculation was performed. The available sample size was determined by the number of eligible participants during the study period.

### Sample size considerations for machine-learning classification

There is no universal minimum sample size applicable to all machine-learning classification algorithms because the required sample size depends on factors including the number and complexity of features, model architecture, outcome prevalence, and the expected signal-to-noise ratio. In the present study, 657 participants were available for analysis, including 298 participants with MetS and 359 with pre-MetS**.** The dataset was subsequently divided using an 80%/20% stratified split, resulting in 525 participants in the training dataset and 132 participants in the independent hold-out test dataset**.** Hyperparameter optimization was performed exclusively within the training dataset using repeated 10-fold cross-validation (10 repetitions), allowing repeated use of the available training data while preserving an independent test dataset for final performance evaluation.

Although the available sample supported the planned comparative classification analyses, the sample size was derived from a single-center retrospective database and may limit model stability and generalizability. Therefore, the resulting models were considered internally validated and exploratory, and external validation in larger, multicenter cohorts is required to determine their robustness and generalizability.

Complete-case analysis was performed because all study variables were required for machine-learning model development**.** No missing-value imputation was performed. To evaluate potential selection bias related to exclusion, baseline demographic and anthropometric characteristics of included and excluded participants were compared using available data, and no statistically significant differences were observed (all *p* > 0.05; S1 Table).

### Definition of pre-metabolic syndrome and metabolic syndrome

Participants were classified using an operational algorithm based on the International Diabetes Federation (IDF) criteria for Asian populations. The complete classification algorithm used to assign participants to the pre-metabolic syndrome (pre-MetS) and metabolic syndrome (MetS) groups is presented in S2 Fig.

MetS was defined as central obesity (waist circumference ≥90 cm in men or ≥80 cm in women) plus at least two of the following components: (i) triglycerides (TG) ≥150 mg/dL; (ii) high-density lipoprotein cholesterol (HDL-C) <40 mg/dL in men or <50 mg/dL in women; (iii) systolic blood pressure (SBP) ≥130 mmHg and/or diastolic blood pressure (DBP) ≥85 mmHg; or (iv) fasting plasma glucose (FPG) ≥100 mg/dL or previously diagnosed type 2 diabetes mellitus (T2DM) [11].

Detailed medication records were not available in the retrospective database. Consequently, MetS classification was based on measured anthropometric, clinical, and biochemical parameters and documented diagnoses of T2DM available in the electronic medical records. Information on antihypertensive and lipid-lowering medication use was unavailable, and treatment-based components of the full IDF definition could therefore not be directly applied. This limitation may have resulted in limited misclassification among participants whose metabolic measurements had been modified by pharmacological treatment.

Pre-MetS was operationally defined as the presence of at least one metabolic abnormality among the IDF-defined components in participants who did not fulfill the complete IDF diagnostic criteria for MetS. Central obesity was not required for classification as pre-MetS. Accordingly, the pre-MetS group included participants with central obesity and fewer than two additional metabolic abnormalities, as well as participants without central obesity who had at least one metabolic abnormality. This operational definition was used to distinguish participants with incomplete manifestations of metabolic syndrome from those meeting the complete IDF diagnostic criteria for MetS.

To improve transparency, the frequencies of the individual MetS components, including central obesity, elevated TG, reduced HDL-C, elevated blood pressure, and elevated FPG or previously diagnosed T2DM, in the pre-MetS and MetS groups are presented in S2 Table.

### Data collection and measurements

Demographic, anthropometric, hemodynamic, biochemical, and metabolic data were collected using standardized protocols.

#### Anthropometric measurements.

Height was measured using a wall-mounted stadiometer (Seca 213, Seca GmbH, Hamburg, Germany). Body weight, total body fat percentage, and visceral fat were measured using bioelectrical impedance analysis (InBody 770, InBody Co., Seoul, Korea) [24]. Waist circumference was measured at the midpoint between the lowest rib and iliac crest using a non-stretchable tape measure, and the average of two measurements was used. Body mass index (BMI) was calculated as weight divided by height squared (kg/m²), and waist-to-height ratio (WHtR) was calculated as waist circumference divided by height [25].

#### Hemodynamic measurements.

Systolic and diastolic blood pressure were measured using an automated digital sphygmomanometer (Omron HEM-907, Omron Healthcare, Kyoto, Japan) after at least 10 minutes of seated rest. The average of two measurements was used for analysis.

#### Biochemical measurements.

Fasting blood samples were collected after an overnight fast of at least 8 hours. Fasting plasma glucose (FPG), triglycerides (TG), total cholesterol, low-density lipoprotein cholesterol (LDL-C), and high-density lipoprotein cholesterol (HDL-C) were measured using enzymatic colorimetric assays. Glycated hemoglobin (HbA1c) was measured using high-performance liquid chromatography, and insulin concentrations were measured using chemiluminescent immunoassays. Uric acid concentrations were measured using standard enzymatic methods.

All laboratory measurements were performed in the central clinical laboratory of HRH Princess Maha Chakri Sirindhorn Medical Center using standardized operating procedures and routine internal and external quality-control programs throughout the study period to ensure measurement consistency.

#### Derived metabolic indices.

Insulin resistance and beta-cell function were estimated using standard equations:

- HOMA-IR = (FPG × insulin)/ 405
- HOMA-β = (360 × insulin)/ (FPG − 63)

Insulin sensitivity was calculated as the reciprocal of HOMA-IR.

Composite metabolic indices included:

- TyG = ln[(TG × FPG)/ 2]
- TyG-BMI = TyG × BMI
- TyG-WC = TyG × waist circumference
- TyG-WHtR = TyG × WHtR
- TG/HDL ratio = TG/ HDL-C
- METS-IR = [ln((2 × FPG) + TG) × BMI]/ ln(HDL-C) [26]

### Statistical analysis and machine learning

A schematic overview of the analytical workflow is shown in S3 Fig.

#### Data preprocessing.

Records were reviewed for completeness before analysis. Participants with missing values for any study variable were excluded; therefore, no missing-value imputation was performed.

Continuous variables were standardized using z-score normalization. To prevent data leakage, normalization parameters, including the mean and standard deviation, were estimated exclusively from the training dataset and subsequently applied unchanged to the independent test dataset.

The dataset was randomly divided into training (80%, *n* = 525) and independent hold-out test (20%, *n* = 132) datasets using stratified random sampling with a fixed random seed (42), thereby preserving the proportion of pre-MetS and MetS participants in both datasets.

The class distribution was relatively balanced, with 54.6% of participants classified as pre-MetS and 45.4% as MetS. Therefore, no synthetic oversampling techniques, including the Synthetic Minority Oversampling Technique (SMOTE), were applied. Such procedures are primarily intended to address substantial class imbalance and were not considered necessary for the present dataset. Accordingly, this study was designed to classify two relatively balanced existing metabolic states rather than to predict a rare future outcome.

#### Feature selection.

Initially, 27 demographic, anthropometric, hemodynamic, biochemical, and derived metabolic variables were considered as candidate features. Feature selection was performed using the Boruta algorithm, an all-relevant feature-selection method based on comparisons between observed predictors and randomized shadow features. The analysis was conducted using the Boruta R package [27] on the training dataset only to prevent information leakage.

Twenty-four features were retained, whereas LDL-C, total cholesterol, and uric acid were classified as rejected by Boruta because their importance was not greater than that of the corresponding shadow features. Complete Boruta results are presented in S4 Fig.

#### Machine-learning model selection and development.

Ten supervised machine-learning algorithms were selected using a structured, tiered approach to provide complementary baseline and nonlinear modeling strategies for tabular clinical data. First, relatively simple and interpretable models, including logistic regression, naïve Bayes, and decision tree, were included as baseline approaches to establish whether the dataset contained a learnable signal and to provide reference points for comparison with more complex algorithms. Logistic regression was selected as the primary conventional linear baseline [28], while naïve Bayes and decision tree provided simple probabilistic and nonlinear tree-based baselines, respectively [29].

Second, k-nearest neighbors, linear support vector machine (SVM), and radial basis function (RBF) SVM were included as classical machine-learning approaches capable of modeling different decision boundaries in structured clinical data. The linear SVM provided a linear margin-based benchmark, whereas the RBF SVM enabled assessment of nonlinear kernel-based relationships [30].

Third, random forest and extreme gradient boosting (XGBoost) were included as tree-based ensemble methods because of their ability to model nonlinear relationships and interactions among predictors and their established applicability to structured/tabular clinical data [31]. Finally, neural network and Gaussian process models were included to evaluate flexible nonlinear and probabilistic modeling approaches [32,33].

This selection represented six major algorithmic families: linear, probabilistic, instance-based, kernel-based, tree-based ensemble, and neural-network/probabilistic nonlinear approaches. The algorithms were selected to provide methodological diversity rather than to prespecify a single expected best-performing model. Comparing these complementary approaches allowed assessment of whether classification performance was consistent across different modeling paradigms for distinguishing pre-MetS from MetS.

Hyperparameters are algorithm-specific settings that control model complexity, learning behavior, and the way predictors contribute to model fitting. Their optimization is important because inappropriate parameter values may result in underfitting or overfitting and may lead to suboptimal or unfair comparisons based on default settings alone. Therefore, hyperparameter optimization was performed to identify an appropriate parameter configuration for each algorithm and to provide a more meaningful comparison of classification performance across different modeling approaches.

Hyperparameter optimization was performed exclusively within the training dataset using grid-search optimization combined with repeated 10-fold cross-validation (10 repetitions). Candidate hyperparameter ranges for each algorithm were predefined based on published recommendations and package documentation. For each algorithm, the optimal hyperparameter combination was selected according to the highest mean cross-validated area under the receiver operating characteristic curve (AUC). Following hyperparameter optimization, final models were trained using the complete training dataset and evaluated using the independent hold-out test dataset. The final optimized hyperparameter settings for all machine-learning algorithms are provided in S3 Table.

#### Model evaluation.

Normality of continuous variables was assessed using the Shapiro-Wilk test. Because several variables were not normally distributed, categorical variables were compared using the chi-square test, whereas continuous variables were compared using the Mann-Whitney U test. Continuous variables are presented as median and interquartile range (IQR).

To complement hypothesis testing, standardized effect sizes were calculated using Cohen’s *d* to quantify the magnitude of between-group differences independently of sample size. Although continuous variables were summarized as medians (IQRs) because of non-normal distributions, Cohen’s *d* was calculated from the original continuous values to facilitate standardized comparisons across variables [34]. Comparisons between the pre-MetS and MetS groups were primarily descriptive and intended to characterize the study population rather than formally test independent hypotheses. Accordingly, individual *p*-values were interpreted cautiously in the context of multiple comparisons, with greater emphasis placed on the magnitude and clinical relevance of observed differences as reflected by standardized effect sizes.

Final classification performance was evaluated using the independent hold-out test dataset (*n* = 132) and summarized using accuracy, sensitivity, specificity, positive predictive value (PPV), negative predictive value (NPV), F1-score, area under the receiver operating characteristic curve (AUC), and Brier score.

AUC was designated as the primary performance metric because it provides a threshold-independent assessment of discrimination and is less influenced by class prevalence than accuracy. Accuracy and F1-score were reported as complementary measures of overall classification performance and the balance between precision and recall.

The probability threshold for binary classification was prespecified at 0.50, corresponding to the conventional default threshold for binary classification and reflecting the relatively balanced class distribution of the study population. This threshold was selected *a priori* and was not optimized using the independent test dataset.

Ninety-five percent confidence intervals for all performance metrics, including AUC, accuracy, sensitivity, specificity, PPV, NPV, F1-score, and Brier score, were estimated using 1,000 bootstrap resamples of the independent hold-out test dataset.

Model calibration was assessed using calibration plots, calibration intercepts, calibration slopes, and Brier scores.

#### Feature importance and model interpretability.

To improve model interpretability, SHAP (Shapley Additive Explanations) analysis was performed for the final XGBoost model. SHAP values were calculated using observations from the independent hold-out test dataset solely for post hoc model interpretation and were not used for model development, feature selection, or hyperparameter optimization.

SHAP values quantify the contribution of each predictor to the model output for individual observations. Feature importance was summarized using the mean absolute SHAP value, which represents the average magnitude of each feature’s contribution to classification regardless of direction. Features with larger mean absolute SHAP values were considered more influential in distinguishing pre-MetS from MetS. SHAP importance was interpreted as model-based contribution rather than evidence of causal or independent biological importance.

#### Sensitivity analysis: exclusion of overlapping composite indices.

Several composite metabolic indices incorporate variables that overlap with components of the International Diabetes Federation (IDF) definition of metabolic syndrome (MetS) and may therefore contribute to optimistic estimates of classification performance. Accordingly, three sensitivity analyses were performed to assess the robustness of the XGBoost model and the potential influence of predictor overlap with the outcome definition.

In Sensitivity analysis 1, the best-performing XGBoost model was retrained after excluding TyG-WC, TyG-WHtR, and METS-IR, which are composite indices incorporating variables that overlap with MetS diagnostic components. This analysis retained 21 predictors.

In Sensitivity analysis 2, all variables directly used in the IDF definition of MetS were excluded, including waist circumference (WC), systolic blood pressure (SBP), diastolic blood pressure (DBP), fasting plasma glucose (FPG), triglycerides (TG), and high-density lipoprotein cholesterol (HDL-C). Derived indices incorporating these diagnostic components, including the TG/HDL-C ratio, TyG, TyG-BMI, TyG-WC, TyG-WHtR, and METS-IR, were also excluded. This analysis retained 12 predictors.

In Sensitivity analysis 3, waist-to-height ratio (WHtR), which incorporates waist circumference, was additionally excluded from the predictor set used in Sensitivity analysis 2. This analysis therefore excluded the MetS diagnostic components, related derived metabolic indices, and WHtR, and retained 11 predictors.

For all sensitivity analyses, the same training/testing split, data preprocessing procedures, feature-selection approach, hyperparameter-tuning strategy, and evaluation metrics used in the primary analysis were applied. The independent hold-out test dataset was not used for feature selection or hyperparameter optimization.

#### Software.

All analyses were performed using R version 4.3.2 (R Foundation for Statistical Computing, Vienna, Austria). Machine-learning analyses were conducted using the caret, xgboost**,** randomForest, e1071, kernlab, nnet, glmnet, pROC, and SHAPforxgboost packages. A fixed random seed (42) was used to facilitate reproducibility.

## Results

### Study population and participant characteristics

A total of 720 individuals were screened for eligibility, of whom 657 met the inclusion criteria and were included in the final analysis, comprising 359 participants with pre-metabolic syndrome (pre-MetS, 54.6%) and 298 participants with metabolic syndrome (MetS, 45.4%). Participant selection is shown in S1 Fig.

Baseline demographic, anthropometric, hemodynamic, biochemical, and metabolic characteristics are summarized in Table 1, which includes the overall study population and both metabolic groups. The overall cohort had a median age of 61.0 years (IQR 52.0–68.0 years), and 63.9% were female. Sex distribution was similar between groups (*p* = 0.740). Participants with MetS generally exhibited higher BMI, waist circumference, waist-to-height ratio, visceral fat, systolic blood pressure, fasting plasma glucose, HbA1c, triglycerides, fasting insulin, HOMA-IR, TyG-derived indices, and METS-IR, together with lower HDL-C than participants with pre-MetS (all *p* < 0.001). Standardized effect sizes (Cohen’s d) ranged from moderate to very large, with the greatest differences observed for visceral fat (d = 1.82), fasting plasma glucose (d = 1.55), waist circumference (d = 1.25), and TyG-WHtR (d = 1.22).

**Table 1:** Baseline characteristics of participants with pre-MetS and MetS.

Parameter	Overall (*n* = 657)	Pre-MetS (*n* = 359)	MetS (*n* = 298)	p-value*	Effect size (Cohen’s d)	IDF component^†^	Composite index^‡^
**Demographics**							
Sex (Male/Female)	237/420	132/227	105/193	0.74	NA	No	No
Age (years)	61.0 (52.0–68.0)	60.0 (51.0–69.0)	62.0 (55.0–68.0)	0.51	0.08	No	No
**Anthropometrics**							
Weight (kg)	68.0 (58.0–80.0)	64.0 (56.0–74.0)	73.0 (64.0–85.0)	<0.001	0.85	No	No
Height (cm)	163.0 (157.0–169.0)	163.0 (157.0–169.0)	162.0 (156.0–168.0)	0.21	−0.15	No	No
BMI (kg/m²)	26.8 (23.5–30.5)	24.9 (22.5–28.1)	29.2 (26.0–32.4)	<0.001	1.12	No	No
Waist circumference^†^ (cm)	88.0 (82.0–96.0)	84.0 (78.0–92.0)	96.0 (90.0–104.0)	<0.001	1.25	Yes	No
WHtR	0.55 (0.52–0.59)	0.53 (0.49–0.57)	0.58 (0.55–0.62)	<0.001	1.18	No	Yes
Total body fat (%)	33.0 (27.5–38.0)	32.0 (26.2–36.8)	34.0 (28.9–39.4)	<0.001	0.45	No	No
Visceral fat	12.0 (7.0–17.0)	9.0 (6.0–14.0)	15.0 (11.0–20.0)	<0.001	1.82	No	No
**Hemodynamics**							
SBP^†^ (mmHg)	128.0 (118.0–138.0)	123.0 (113.0–133.0)	133.0 (124.0–142.0)	<0.001	0.72	Yes	No
DBP^†^ (mmHg)	78.0 (69.0–85.0)	77.0 (68.0–86.0)	78.0 (70.0–85.0)	0.91	0.03	Yes	No
**Biochemical variables**							
FPG^†^ (mg/dL)	115.0 (95.0–135.0)	95.0 (85.0–105.0)	135.0 (120.0–150.0)	<0.001	1.55	Yes	No
HbA1c (%)	5.9 (5.5–6.4)	5.6 (5.4–5.9)	6.3 (5.8–6.9)	<0.001	1.12	No	No
Insulin (µIU/mL)	13.0 (9.0–20.0)	10.6 (7.9–16.7)	17.1 (12.1–25.7)	<0.001	0.92	No	No
HOMA-IR	3.5 (2.4–5.6)	2.8 (2.1–4.2)	5.0 (3.3–7.6)	<0.001	1.06	No	No
HOMA-β	100.0 (45.0–160.0)	94.8 (30.3–145.9)	115.1 (76.2–178.7)	<0.001	0.48	No	No
Insulin sensitivity	0.28 (0.18–0.42)	0.36 (0.24–0.48)	0.20 (0.13–0.30)	<0.001	−1.14	No	No
**Lipids**							
TG^†^ (mg/dL)	120.0 (85.0–165.0)	105.0 (80.0–150.0)	140.0 (100.0–180.0)	<0.001	0.68	Yes	No
HDL-C^†^ (mg/dL)	49.0 (41.0–60.0)	52.0 (43.0–63.0)	46.0 (40.0–56.0)	<0.001	−0.55	Yes	No
**Composite metabolic indices**							
TyG index	8.8 (8.5–9.2)	8.7 (8.3–9.1)	8.9 (8.6–9.3)	<0.001	0.62	No	Yes
TyG-WC	800.0 (710.0–890.0)	765.0 (680.0–845.0)	856.0 (780.0–930.0)	<0.001	0.98	No	Yes
TyG-BMI	230.0 (200.0–265.0)	210.0 (185.0–240.0)	255.0 (225.0–290.0)	<0.001	1.15	No	Yes
TyG-WHtR	5.0 (4.6–5.5)	4.7 (4.3–5.1)	5.4 (5.0–5.8)	<0.001	1.22	No	Yes
TG/HDL-C ratio	2.3 (1.6–3.5)	1.9 (1.4–2.9)	3.0 (2.0–4.6)	<0.001	0.82	No	Yes
METS-IR	40.0 (35.0–47.0)	37.0 (32.0–42.0)	46.0 (41.0–52.0)	<0.001	1.18	No	Yes
**Other variables**							
Uric acid (mg/dL)	5.9 (5.0–6.8)	5.6 (4.7–6.5)	6.3 (5.4–7.1)	<0.001	0.72	No	No
Total cholesterol (mg/dL)	180.0 (158.0–208.0)	170.0 (150.0–200.0)	190.0 (168.0–215.0)	<0.001	0.62	No	No
LDL-C (mg/dL)	108.0 (86.0–131.0)	102.0 (80.0–125.0)	115.0 (95.0–138.0)	<0.001	0.58	No	No

Data are presented as median (interquartile range) unless otherwise indicated. Continuous variables were compared using the Mann-Whitney U test and categorical variables using the chi-square test. Effect size was quantified using Cohen’s d and interpreted using conventional thresholds: small (0.20–0.49), medium (0.50–0.79), and large (≥0.80).

†IDF diagnostic components**:** Variables included directly in the International Diabetes Federation (IDF) definition of metabolic syndrome. Because these variables contribute directly to outcome classification, between-group differences are expected and should be interpreted as descriptive characteristics of the classification scheme rather than independent confirmatory findings.

‡Composite indices**:** Variables mathematically derived from one or more metabolic parameters included in the IDF definition (e.g., waist circumference, triglycerides, fasting plasma glucose, or HDL-C). Consequently, observed group differences may partly reflect overlap with the outcome definition.

Abbreviations: BMI, body mass index; DBP, diastolic blood pressure; FPG, fasting plasma glucose; HbA1c, glycated hemoglobin; HDL-C, high-density lipoprotein cholesterol; HOMA-IR, homeostasis model assessment of insulin resistance; HOMA-β, homeostasis model assessment of β-cell function; IDF, International Diabetes Federation; LDL-C, low-density lipoprotein cholesterol; MetS, metabolic syndrome; METS-IR, metabolic score for insulin resistance; pre-MetS, pre-metabolic syndrome; SBP, systolic blood pressure; TG, triglycerides; TyG, triglyceride-glucose index; TyG-BMI, triglyceride-glucose body mass index; TyG-WC, triglyceride-glucose waist circumference; TyG-WHtR, triglyceride-glucose waist-to-height ratio; WHtR, waist-to-height ratio.

Because waist circumference, blood pressure, fasting plasma glucose, triglycerides, and HDL-C are components of the International Diabetes Federation (IDF) diagnostic criteria for MetS, the observed between-group differences for these variables were expected and should be interpreted as descriptive baseline characteristics rather than independent confirmatory findings.

The distribution of individual MetS diagnostic components is presented in S2 Table. As expected, participants with MetS more frequently exhibited central obesity, elevated triglycerides, reduced HDL-C, elevated blood pressure, and elevated fasting plasma glucose or previously diagnosed type 2 diabetes than participants with pre-MetS. These descriptive results are consistent with the operational definitions used for metabolic classification.

### Balance between training and testing datasets

Following stratified random sampling, 525 participants (80%) were allocated to the training dataset and 132 participants (20%) to the independent testing dataset. Comparisons between the two datasets demonstrated no statistically significant differences in age, sex distribution, BMI, waist circumference, systolic blood pressure, diastolic blood pressure, fasting plasma glucose, triglycerides, HDL-C, or the prevalence of MetS (all *p* > 0.05), indicating that stratified sampling successfully preserved the distribution of demographic and metabolic characteristics (S4 Table).

### Boruta feature selection

Twenty-seven candidate variables, encompassing demographic, anthropometric, hemodynamic, biochemical, and derived metabolic measures, were evaluated using the Boruta algorithm in the training dataset. Twenty-four variables were confirmed as important and retained for model development, whereas LDL-C, total cholesterol, and uric acid were rejected because their importance scores did not exceed those of the corresponding shadow features (S4 Fig). The retained variables were subsequently used for machine-learning model development and evaluation.

### Machine learning classification performance

The classification performance of the ten machine-learning (ML) algorithms was evaluated using an independent testing dataset (*n* = 132). Performance metrics, including accuracy, sensitivity, specificity, positive predictive value (PPV), negative predictive value (NPV), F1-score, area under the receiver operating characteristic curve (AUC), and the Brier score, together with their 95% confidence intervals, are summarized in Table 2. For all performance metrics, MetS was designated as the positive class. Receiver operating characteristic (ROC) curves for the top-performing models are shown in Fig 1, whereas ROC curves for all evaluated models are presented in S5 Fig.

**Table 2:** Performance of ML models for classification of pre-MetS and MetS.

Model	AUC	Accuracy	Sensitivity	Specificity	PPV	NPV	F1-Score	Brier Score
XGBoost	0.986 (0.970–0.997)	0.932 (0.885–0.969)	0.882 (0.792–0.958)	0.973 (0.930–1.000)	0.965 (0.906–1.000)	0.909 (0.835–0.964)	0.921 (0.864–0.967)	0.050 (0.026–0.080)
Neural Network	0.948 (0.906–0.983)	0.878 (0.823–0.931)	0.815 (0.712–0.908)	0.930 (0.868–0.985)	0.906 (0.824–0.978)	0.859 (0.779–0.928)	0.857 (0.786–0.922)	0.085 (0.053–0.122)
Random Forest	0.942 (0.905–0.974)	0.886 (0.831–0.938)	0.833 (0.733–0.924)	0.931 (0.862–0.985)	0.910 (0.830–0.981)	0.869 (0.787–0.940)	0.869 (0.800–0.930)	0.101 (0.073–0.129)
Gaussian Process	0.920 (0.868–0.960)	0.815 (0.746–0.877)	0.744 (0.625–0.850)	0.874 (0.789–0.947)	0.830 (0.725–0.925)	0.805 (0.712–0.885)	0.783 (0.692–0.862)	0.125 (0.098–0.154)
Logistic Regression	0.916 (0.867–0.959)	0.808 (0.738–0.877)	0.730 (0.607–0.845)	0.874 (0.789–0.946)	0.828 (0.711–0.925)	0.795 (0.704–0.880)	0.775 (0.680–0.857)	0.119 (0.083–0.158)
k-Nearest Neighbors	0.906 (0.853–0.950)	0.808 (0.738–0.877)	0.763 (0.652–0.867)	0.845 (0.754–0.924)	0.803 (0.691–0.907)	0.811 (0.714–0.899)	0.781 (0.694–0.857)	0.124 (0.093–0.159)
Naïve Bayes	0.882 (0.821–0.933)	0.769 (0.692–0.838)	0.761 (0.648–0.869)	0.774 (0.676–0.873)	0.738 (0.625–0.851)	0.795 (0.697–0.889)	0.748 (0.654–0.831)	0.204 (0.138–0.272)
Decision Tree	0.868 (0.799–0.929)	0.848 (0.785–0.908)	0.783 (0.671–0.878)	0.901 (0.827–0.962)	0.867 (0.768–0.948)	0.834 (0.742–0.908)	0.822 (0.733–0.891)	0.132 (0.086–0.183)
Linear SVM	0.818 (0.749–0.882)	0.824 (0.754–0.885)	0.763 (0.650–0.866)	0.874 (0.791–0.952)	0.835 (0.723–0.933)	0.816 (0.728–0.897)	0.796 (0.708–0.872)	0.146 (0.109–0.188)
RBF SVM	0.804 (0.731–0.869)	0.809 (0.738–0.869)	0.748 (0.630–0.855)	0.860 (0.775–0.930)	0.816 (0.706–0.909)	0.804 (0.708–0.892)	0.779 (0.688–0.857)	0.155 (0.118–0.197)

**Notes:** Values are presented as performance estimates with 95% confidence intervals (95% CIs). Hyperparameter tuning was performed using repeated 10-fold cross-validation with ten repetitions within the training dataset. Final classification performance was evaluated in the independent hold-out test dataset. Ninety-five percent confidence intervals for all performance metrics were estimated using 1,000 bootstrap resamples. MetS was designated as the positive class. Models are ranked according to AUC. A probability threshold of 0.50 was used for binary classification. Lower Brier scores indicate better calibration.

Abbreviations: AUC, area under the receiver operating characteristic curve; CI, confidence interval; kNN, k-nearest neighbors; MetS, metabolic syndrome; NPV, negative predictive value; PPV, positive predictive value; pre-MetS, pre-metabolic syndrome; RBF SVM, radial basis function support vector machine; XGBoost, extreme gradient boosting.

**Fig 1:**
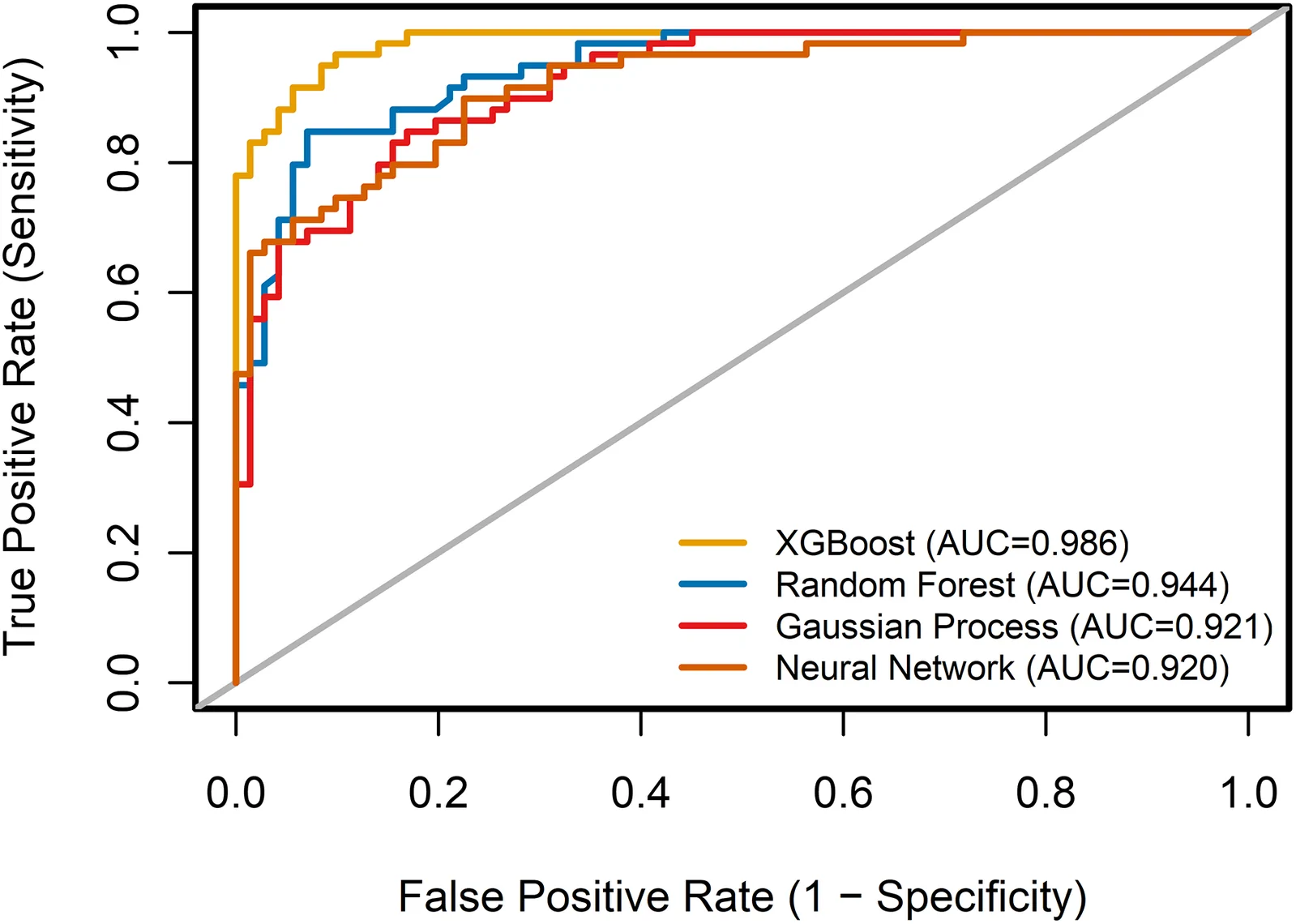
ROC curves of the top-performing machine-learning models for classification of pre-MetS and MetS. ROC curves were generated using the independent hold-out test dataset (*n* = 132). XGBoost demonstrated the highest discrimination (AUC = 0.986), followed by Neural Network (AUC = 0.948), Random Forest (AUC = 0.942), and Gaussian Process (AUC = 0.920). The dashed diagonal line represents no discrimination (AUC = 0.5). **Abbreviations:** AUC, area under the receiver operating characteristic curve; ROC, receiver operating characteristic; XGBoost, extreme gradient boosting.

Overall, model discrimination varied across the evaluated algorithms, with AUC values ranging from 0.804 to 0.986. Brier scores ranged from 0.050 to 0.204, indicating substantial variability in discrimination and calibration performance across models. Tree-based ensemble methods consistently achieved the highest overall performance, whereas support vector machine and naïve Bayes models generally demonstrated lower discrimination and poorer calibration.

Among all evaluated algorithms, XGBoost achieved the highest classification performance. It demonstrated the highest accuracy (0.932, 95% CI: 0.885–0.969), specificity (0.973, 95% CI: 0.930–1.000), positive predictive value (0.965, 95% CI: 0.906–1.000), F1-score (0.921, 95% CI: 0.864–0.967), and AUC (0.986, 95% CI: 0.970–0.997), together with the lowest Brier score (0.050, 95% CI: 0.026–0.080) among all evaluated models (Table 2).

Calibration analysis of the XGBoost model yielded a calibration intercept of 0.71 (95% CI: −0.02 to 1.39), a calibration slope of 1.30 (95% CI: 1.01–2.41), and a Brier score of 0.050 (95% CI: 0.026–0.080). The calibration plot demonstrated reasonable agreement between predicted probabilities and observed event rates across most risk strata, although relatively wide confidence intervals were observed in several groups because of the limited sample size of the independent test dataset (*n* = 132) (Fig 2).

**Fig 2:**
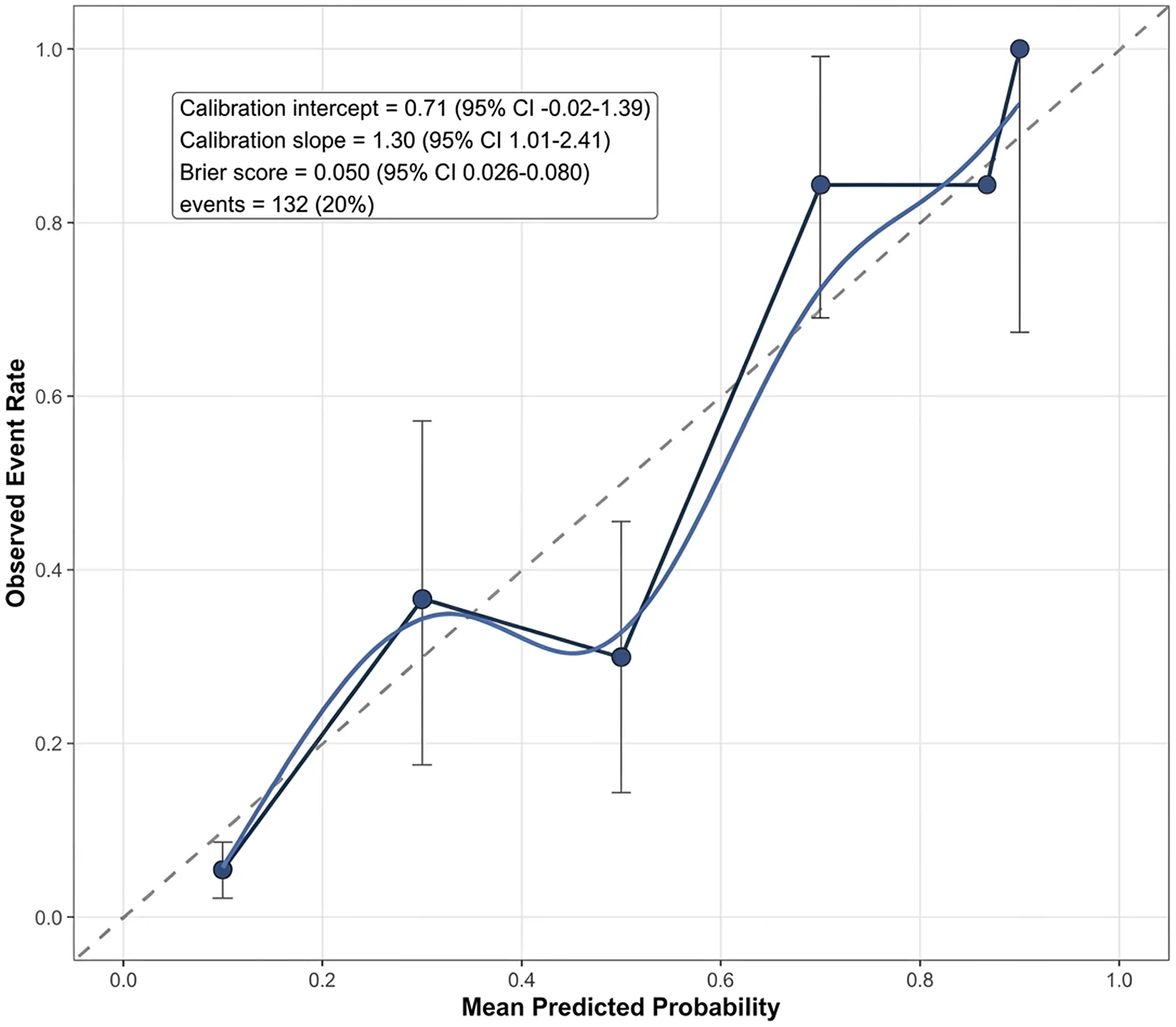
Calibration plot of the XGBoost model in the independent test dataset. Observed event rates are plotted against mean predicted probabilities across grouped risk strata (*n* = 132). The dashed diagonal line indicates perfect calibration. Points represent observed event rates, and error bars indicate 95% confidence intervals. The calibration intercept was 0.71 (95% CI: −0.02 to 1.39), the calibration slope was 1.30 (95% CI: 1.01-2.41), and the Brier score was 0.050 (95% CI: 0.026-0.080). **Abbreviation:** XGBoost, extreme gradient boosting.

These findings indicate that XGBoost achieved the highest classification performance among the evaluated models. However, interpretation of these findings should consider that several predictors overlapped with components of the International Diabetes Federation (IDF) definition of metabolic syndrome. Consequently, part of the observed classification performance may be attributable to overlap between predictor variables and the diagnostic criteria used to define the outcome.

### Feature importance

SHAP analysis identified systolic blood pressure (SBP) as the most influential predictor in the final XGBoost model, followed by fasting plasma glucose (FPG), waist circumference (WC), sex, and TyG-WHtR (Fig 3). Additional influential features included HDL-C, TyG-WC, triglycerides (TG), the TG/HDL-C ratio, and waist-to-height ratio (WHtR).

**Fig 3:**
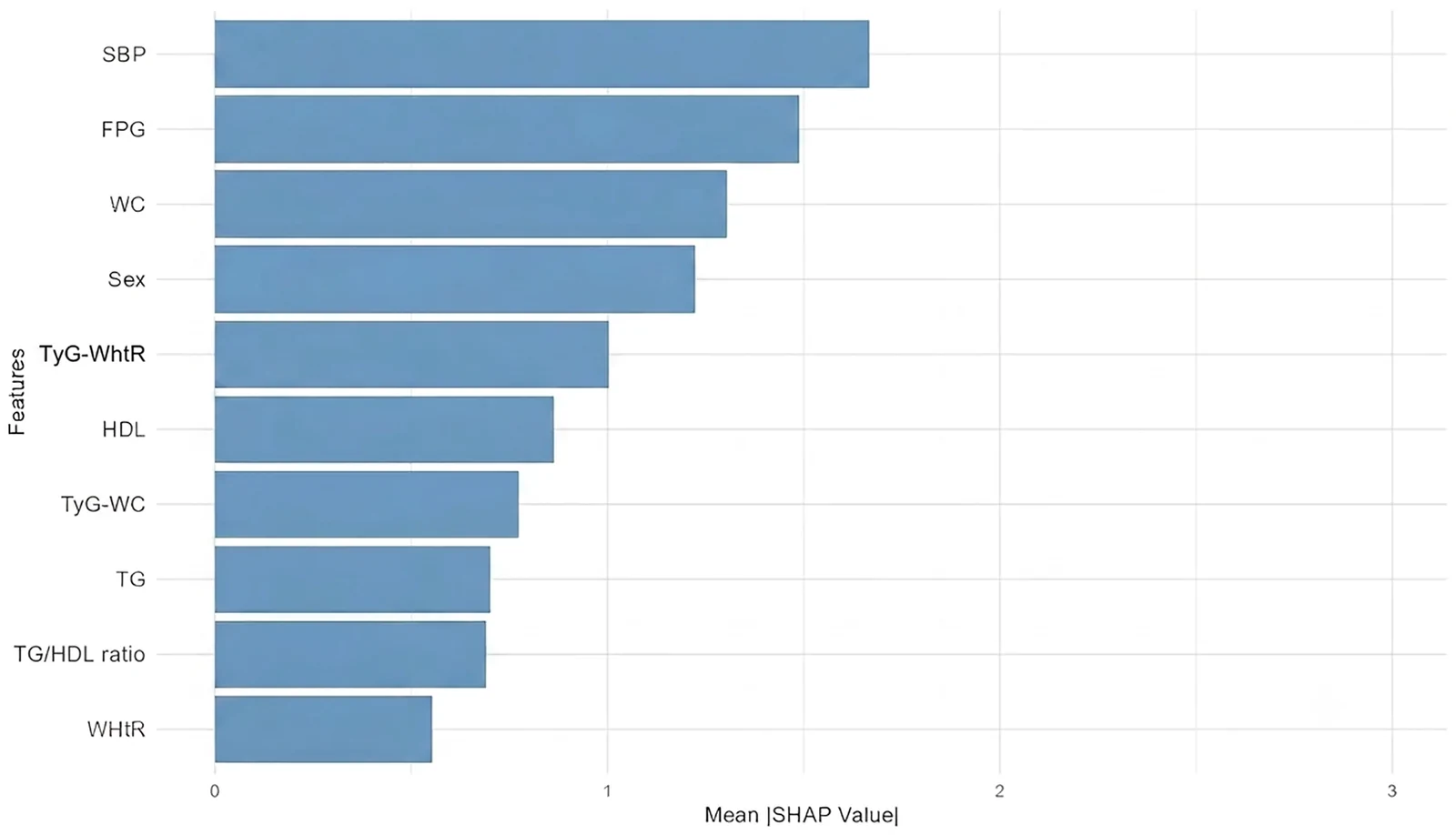
SHAP feature importance of the final XGBoost model for classification of pre-MetS and MetS. The bar chart shows the mean absolute SHAP (Shapley Additive Explanations) values of the 10 most influential features in the final XGBoost model. Higher mean absolute SHAP values indicate a greater average contribution of a feature to model classification. Several highly ranked features are components of the International Diabetes Federation (IDF) definition of metabolic syndrome or are derived from those components; therefore, feature-importance rankings should be interpreted with caution. **Abbreviations:** DBP, diastolic blood pressure; FPG, fasting plasma glucose; HDL-C, high-density lipoprotein cholesterol; SBP, systolic blood pressure; SHAP, Shapley Additive Explanations; TG, triglycerides; TyG-WC, triglyceride-glucose waist circumference; TyG-WHtR, triglyceride-glucose waist-to-height ratio; WC, waist circumference; XGBoost, extreme gradient boosting.

The highest mean absolute SHAP values were observed for SBP, FPG, and WC, indicating that these variables contributed most strongly to model classification. Several highly ranked features overlap directly with components of the International Diabetes Federation (IDF) diagnostic criteria for metabolic syndrome or are derived from those components. Therefore, the observed feature-importance rankings should be interpreted cautiously because part of their contribution may reflect overlap with the outcome definition rather than entirely independent discriminatory information.

### Sensitivity analysis

To evaluate the influence of feature overlap with the International Diabetes Federation (IDF) diagnostic components for metabolic syndrome (MetS), three sensitivity analyses were performed using the XGBoost model (S5 Table).

In the primary analysis, which included all 24 retained features, XGBoost achieved excellent classification performance with an AUC of 0.986 (95% CI: 0.970–0.997), an accuracy of 0.932 (95% CI: 0.885–0.969), and a Brier score of 0.050 (95% CI: 0.026–0.080).

In Sensitivity Analysis 1, composite indices incorporating MetS diagnostic components (TyG-WC, TyG-WHtR, and METS-IR) were excluded, leaving 21 features. Model performance decreased but remained good, with an AUC of 0.912 (95% CI: 0.878–0.946), an accuracy of 0.847 (95% CI: 0.801–0.885), and a Brier score of 0.094 (95% CI: 0.064–0.134).

In Sensitivity Analysis 2, all variables directly included in the IDF definition of MetS (waist circumference, systolic blood pressure, diastolic blood pressure, fasting plasma glucose, triglycerides, and HDL-C), together with related derived indices, were excluded, leaving 12 non-diagnostic features. Model performance declined further, with an AUC of 0.875 (95% CI: 0.836–0.914), an accuracy of 0.792 (95% CI: 0.742–0.835), and a Brier score of 0.142 (95% CI: 0.106–0.184).

In Sensitivity Analysis 3, waist-to-height ratio (WHtR), which incorporates waist circumference, was additionally excluded, leaving 11 features. Although performance decreased further, the model retained acceptable discriminatory ability, achieving an AUC of 0.861 (95% CI: 0.819–0.903), an accuracy of 0.776 (95% CI: 0.724–0.822), and a Brier score of 0.158 (95% CI: 0.120–0.202).

Overall, classification performance declined progressively as variables directly related to the diagnostic definition of MetS were removed. The marked reduction in discrimination from an AUC of 0.986 in the primary analysis to 0.861 in the most stringent sensitivity analysis suggests that overlap between predictor variables and the MetS diagnostic criteria contributed substantially to the performance of the primary model. Nevertheless, the retention of acceptable discrimination after exclusion of all diagnostic components and related derived variables indicates that non-diagnostic clinical and metabolic characteristics continued to contribute to classification of metabolic status.

## Discussion

### Principal findings

In this retrospective cross-sectional study of Thai adults, we developed and compared ten supervised machine-learning (ML) algorithms for classifying existing pre-metabolic syndrome (pre-MetS) and metabolic syndrome (MetS) using routinely collected demographic, anthropometric, clinical, and biochemical variables. Among the evaluated algorithms, XGBoost achieved the highest classification performance in the independent hold-out test dataset, followed by the neural network and random forest models, whereas conventional linear approaches and support vector machine models demonstrated comparatively lower discrimination. XGBoost achieved an AUC of 0.986 (95% CI: 0.970–0.997), an accuracy of 0.932, and the lowest Brier score among all evaluated algorithms.

The present study successfully addressed all predefined objectives. First, significant differences in anthropometric, hemodynamic, biochemical, and metabolic characteristics were observed between participants with pre-MetS and MetS. Second, ten ML algorithms were systematically evaluated, demonstrating substantial variability in classification performance across modeling approaches. Third, SHAP analysis identified systolic blood pressure (SBP), fasting plasma glucose (FPG), waist circumference (WC), sex, TyG-WHtR, HDL-C, TyG-WC, triglycerides (TG), the TG/HDL-C ratio, and waist-to-height ratio (WHtR) as the most influential contributors to model classification.

Among the evaluated algorithms, XGBoost consistently achieved the highest performance across multiple metrics, including AUC, accuracy, F1-score, and Brier score. These findings suggest that ensemble tree-based methods may be particularly well suited for modeling the complex and potentially nonlinear interactions among clinical and metabolic variables involved in metabolic dysfunction. The SHAP analysis further demonstrated that SBP, WC, and FPG were the dominant contributors to classification, findings that remained broadly consistent with the results of the sensitivity analyses.

The findings demonstrate the feasibility of applying ML to classify existing metabolic states using routinely available clinical data. However, the purpose of ML in this study was not to replace the established International Diabetes Federation (IDF) diagnostic criteria or to predict future disease progression. Rather, ML was evaluated as a complementary analytical approach to determine how multiple routinely collected variables collectively distinguish individuals with pre-MetS from those with established MetS.

Importantly, the exceptionally high discrimination achieved by the primary XGBoost model should be interpreted cautiously. Sensitivity analyses demonstrated that exclusion of overlapping composite indices reduced the AUC from 0.986 to 0.912, indicating that these derived markers contributed meaningfully to model performance. Further exclusion of all variables directly included in the IDF definition of MetS and related derived indices reduced the AUC to 0.875, while additional exclusion of WHtR resulted in an AUC of 0.861. These findings suggest that a substantial proportion of the discrimination observed in the primary model was attributable to variables overlapping with, or closely related to, the diagnostic definition of MetS. Nevertheless, the model retained acceptable discriminatory ability after removal of these variables, indicating that broader clinical and metabolic characteristics also contributed to classification performance.

### Diagnostic circularity and interpretation of model performance

The most important consideration when interpreting the present findings is diagnostic circularity. Several predictors included in the primary feature set directly define MetS according to the IDF criteria, including WC, FPG, TG, HDL-C, SBP, and DBP. In addition, composite indices such as TyG-WC, TyG-WHtR, TG/HDL-C ratio, and METS-IR incorporate some of these same variables. Consequently, part of the observed discrimination likely reflects reconstruction of the diagnostic rule rather than identification of entirely independent metabolic characteristics.

The SHAP analysis supports this interpretation. SBP, WC, and FPG were among the most influential features, and nine of the ten most important variables were either direct IDF diagnostic components or indices derived from those components. Their high SHAP values therefore indicate strong model reliance for classification but should not be interpreted as evidence of independent biological importance. Moreover, correlations among related metabolic variables may influence the allocation of SHAP importance across features.

The sensitivity analyses provided a quantitative evaluation of potential diagnostic circularity. Removal of TyG-WC, TyG-WHtR, and METS-IR reduced the AUC from 0.986 to 0.912, indicating that these composite indices contributed appreciably to model discrimination. When all variables directly included in the IDF definition of MetS, together with related derived indices, were excluded, the AUC decreased further to 0.875. Additional exclusion of WHtR reduced the AUC to 0.861 and increased the Brier score from 0.050 to 0.158. Collectively, these findings indicate that overlap with the diagnostic definition contributed substantially to the high performance observed in the primary analysis.

Nevertheless, the persistence of an AUC of 0.861 after exclusion of all MetS diagnostic components, related derived indices, and WHtR indicates that discrimination was not entirely dependent on variables directly related to the IDF diagnostic criteria. Rather, the remaining routinely collected anthropometric, biochemical, and metabolic variables appeared to provide additional information for distinguishing pre-MetS from MetS. However, because features and outcomes were measured concurrently in a cross-sectional dataset, these variables should be interpreted as correlates of existing metabolic status rather than as independent features of future progression from pre-MetS to MetS.

### Comparison with previous machine-learning studies

The finding that XGBoost and other tree-based ensemble approaches achieved the strongest classification performance is broadly consistent with previous ML studies evaluating MetS and related cardiometabolic conditions [9,35]. Ensemble methods, including random forest and gradient boosting algorithms, have frequently demonstrated superior discrimination because they can effectively accommodate nonlinear relationships, complex interactions, and heterogeneous predictor effects.

In the present study, XGBoost outperformed logistic regression and support vector machine models, suggesting that nonlinear modeling approaches may provide advantages when distinguishing closely related metabolic states. A possible explanation is that metabolic dysfunction arises from multiple interacting physiological processes that may not be adequately represented by simpler linear models.

Direct comparison of discrimination metrics across studies should be interpreted cautiously. Previous ML investigations have differed considerably in study populations, diagnostic definitions, sample sizes, predictor variables, and validation strategies. Some studies incorporated lifestyle factors, dietary information, imaging data, or genetic markers, whereas the present study focused primarily on routinely available clinical and biochemical variables [36,37].

Another important distinction relates to the classification task itself. Many previous ML studies compared individuals with MetS against metabolically healthy controls, whereas our study focused on distinguishing pre-MetS from MetS [38–40]. Because both groups already exhibit metabolic abnormalities, this represents a more challenging and clinically relevant classification problem. Consequently, reported performance metrics should not be directly compared across these fundamentally different classification tasks.

A further distinction of the present study is the explicit evaluation of predictor-outcome overlap. Few previous ML studies have quantified the extent to which classification performance may be driven by variables incorporated within the diagnostic definition of MetS [38,41]. Our sensitivity analyses demonstrated that although discrimination remained high after exclusion of overlapping composite indices, substantial reductions were observed when diagnostic components themselves were removed.

Because few ML studies have specifically evaluated feature importance for classification of pre-MetS versus MetS, conventional epidemiological and pathophysiological studies were used primarily to assess the biological plausibility of the identified features rather than to compare predictive performance [42,43]. Therefore, references to non-ML studies should be interpreted as providing biological context rather than methodological comparison.

Overall, the findings support previous evidence suggesting that tree-based ensemble approaches perform particularly well when applied to structured metabolic datasets while also highlighting the importance of evaluating diagnostic circularity when ML is used to classify clinically defined syndromes.

### Biological interpretation of feature importance

SHAP analysis identified SBP, FPG, WC, sex, TyG-WHtR, HDL-C, TyG-WC, TG, the TG/HDL-C ratio, and WHtR as the most influential variables in the final XGBoost model. These findings are clinically plausible because central adiposity, dysglycemia, dyslipidemia, and elevated blood pressure are core characteristics of metabolic dysfunction and are closely related to the pathophysiology of MetS [44–46]. However, interpretation of feature importance requires caution. Several highly ranked variables are components of the IDF diagnostic criteria or are mathematically derived from those components. Consequently, high SHAP values indicate that the model relied heavily on these variables for classification but should not be interpreted as evidence that they are independent determinants of metabolic syndrome. Similarly, the high importance of TyG-derived indices likely reflects their incorporation of variables already closely related to the MetS definition.

The persistence of meaningful discrimination after removal of diagnostic variables suggests that broader metabolic and anthropometric characteristics also contribute to classification. Nevertheless, because the study was cross-sectional, these variables should be interpreted as correlates of existing metabolic status rather than predictors of future progression from pre-MetS to MetS.

### Implications and justification for machine-learning application

The principal implication of this study is that ML can integrate routinely collected clinical and biochemical variables to classify individuals across closely related stages of metabolic dysfunction. The use of multiple algorithms enabled evaluation of whether classification performance depended on the underlying modeling approach and demonstrated that flexible nonlinear methods outperformed more conventional linear approaches.

A possible explanation for the superior performance of XGBoost is its ability to model complex nonlinear relationships and interactions among predictors while incorporating regularization mechanisms that help limit overfitting. In contrast, simpler models may be less capable of capturing the multidimensional nature of metabolic dysfunction.

However, the present findings do not demonstrate that ML is superior to the IDF criteria for diagnosing MetS. Indeed, part of the observed performance resulted from the inclusion of variables directly related to the diagnostic definition. Therefore, the primary contribution of ML in this study lies in characterizing how multiple routinely measured variables collectively contribute to metabolic classification rather than replacing conventional diagnostic approaches.

The retention of meaningful discrimination after removal of diagnostic components provides a rationale for future ML investigations using predictor sets that minimize overlap with outcome definitions. Such studies could determine whether non-diagnostic clinical, biochemical, behavioral, or environmental variables provide incremental information beyond existing diagnostic frameworks.

Accordingly, the present findings should be considered hypothesis-generating. Future research should directly compare ML models with rule-based diagnostic systems, assess incremental predictive value, evaluate decision-making impact, and determine whether ML can identify clinically meaningful information beyond the variables already incorporated into established diagnostic criteria.

### Clinical implications

The models developed in this study should be interpreted as classification tools rather than prognostic models. Because the study employed a retrospective cross-sectional design, the models classify existing metabolic status and cannot predict future progression from pre-MetS to MetS or subsequent cardiovascular and diabetic outcomes.

From a clinical perspective, the strong performance of XGBoost demonstrates that routinely collected clinical variables contain sufficient information to distinguish between pre-MetS and MetS within this cohort. However, whether implementation of such a model would provide meaningful benefit beyond the established IDF criteria remains uncertain.

The potential clinical role of ML is therefore likely to be complementary rather than substitutive. Future studies should investigate whether ML can integrate additional non-diagnostic variables and identify metabolic patterns that are not fully captured by threshold-based diagnostic systems. Before clinical implementation, external validation, calibration assessment, comparison with conventional diagnostic approaches, and evaluation of clinical utility through decision-curve analysis will be required.

### Strengths and limitations

This study has several strengths. First, ten supervised machine-learning algorithms representing diverse analytical approaches were evaluated using a standardized modeling framework, with logistic regression serving as a conventional baseline and additional classical, kernel-based, tree-based ensemble, and nonlinear approaches providing complementary comparisons. Second, feature selection using the Boruta algorithm, data normalization, hyperparameter optimization, and model training were performed exclusively within the training dataset, thereby minimizing information leakage. Third, model performance was evaluated using an independent hold-out test dataset, with comprehensive assessment of discrimination and calibration, including AUC, accuracy, sensitivity, specificity, predictive values, F1-score, calibration plots, calibration intercepts, calibration slopes, and Brier scores. Fourth, sensitivity analyses explicitly evaluated the influence of overlap between predictor variables and the IDF diagnostic criteria, providing a more transparent assessment of potential diagnostic circularity. Although model performance declined substantially following progressive exclusion of overlapping predictors, the retention of an AUC of 0.861 in the most stringent sensitivity analysis indicates that discrimination was not entirely dependent on variables directly related to the diagnostic criteria. This finding suggests that non-diagnostic clinical and metabolic variables also contributed to model classification. Finally, this study contributes evidence from a Thai population to the relatively limited literature on machine-learning-based metabolic classification in Southeast Asia.

Several limitations should also be acknowledged. Most importantly, diagnostic circularity may have contributed substantially to the high classification performance of the primary models because several features were components of the IDF definition of MetS or were derived from those components. The SHAP analysis identified SBP, WC, and FPG among the most influential features, further highlighting this overlap. Although sensitivity analyses progressively excluded overlapping diagnostic components and related derived indices and demonstrated reduced discrimination, residual dependence between predictors and the outcome definition cannot be completely excluded.

Second, the retrospective cross-sectional design precludes causal inference and does not permit prediction of progression from pre-MetS to MetS or future cardiometabolic outcomes. The models should therefore be interpreted as classifiers of existing metabolic status rather than prognostic models. Third, participants were recruited from a single tertiary-care medical center, which may limit the generalizability and transportability of the findings to other healthcare settings and populations. Fourth, complete-case analysis was used, and participants with incomplete data were excluded. Although no statistically significant differences were observed in the available baseline characteristics between included and excluded participants, this does not exclude residual selection bias.

Fifth, detailed medication information was unavailable. Consequently, treatment-related components of the full IDF definition could not be directly incorporated, and pharmacological treatment may have modified measured metabolic parameters, potentially resulting in some degree of outcome misclassification. Sixth, although the available sample supported the planned comparative classification analyses, the 657 participants were derived from a single-center retrospective database and the independent hold-out test set comprised only 132 participants. Thus, estimates of model performance may remain uncertain, particularly for individual performance metrics. Finally, external validation was not performed. The reproducibility, calibration, and transportability of the models therefore remain to be established in larger, independent, multicenter populations before clinical implementation can be considered.

## Conclusion

In this retrospective cross-sectional study of Thai adults, machine-learning models, particularly XGBoost, demonstrated high classification performance for distinguishing existing pre-metabolic syndrome (pre-MetS) from metabolic syndrome (MetS) using routinely available clinical and biochemical variables in a single-center cohort with an independent hold-out test set. The models were developed to classify current metabolic status rather than predict future disease progression; therefore, further prospective longitudinal studies are needed to determine whether these classification patterns have predictive value for future metabolic outcomes.

SHAP analysis identified systolic blood pressure (SBP), fasting plasma glucose (FPG), waist circumference (WC), sex, TyG-WHtR, HDL-C, TyG-WC, triglycerides (TG), TG/HDL-C ratio, and WHtR as the most influential features. However, these findings should be interpreted with substantial caution because several influential features are components of, or are derived from variables included in, the International Diabetes Federation (IDF) diagnostic criteria for MetS. Thus, part of the observed classification performance likely reflects overlap with the outcome definition rather than entirely independent metabolic signatures, as supported by the reduced discrimination observed in sensitivity analyses.

Accordingly, these findings should be considered exploratory and require external validation. Although the results suggest that routinely collected clinical and biochemical variables contain information relevant to metabolic status, whether machine-learning approaches provide clinically meaningful benefit beyond established IDF diagnostic criteria remains uncertain. The single-center design may also limit the generalizability of the findings to other healthcare settings and populations. Multicenter prospective studies incorporating predictor sets that minimize overlap with diagnostic criteria, comprehensive medication data, external validation, calibration assessment, and decision-curve analysis are needed before clinical implementation can be considered.

## Supporting information

S1 Fig Study Population and Data Preparation. (PDF)

S2 Fig Classification algorithm used to assign participants to pre-metabolic syndrome (pre-MetS) and metabolic syndrome (MetS) groups. (PDF)

S3 Fig Machine Learning Model Development and Evaluation. (PDF)

S4 Fig Boruta feature selection: median importance scores and variable classification across candidate features. (PDF)

S5 Fig Receiver operating characteristic (ROC) curves of machine learning models for classification of pre-metabolic syndrome (pre-MetS) and metabolic syndrome (MetS). (PDF)

S1 Table Comparison of baseline characteristics between included and excluded participants. (PDF)

S2 Table Frequency of individual metabolic syndrome diagnostic components in pre-MetS and MetS groups. (PDF)

S3 Table Hyperparameter search grids and final selected parameters for machine-learning models. (PDF)

S4 Table Baseline characteristics of participants in the training and test datasets. (PDF)

S5 Table Sensitivity analyses of XGBoost performance after exclusion of overlapping and MetS-related features. (PDF)

## Abbreviations

AUC: area under the receiver operating characteristic curve
BMI: body mass index
CI: confidence interval
FPG: fasting plasma glucose
HbA1c: glycated hemoglobin
HDL-C: high-density lipoprotein cholesterol
HOMA-β: homeostatic model assessment of beta-cell function
HOMA-IR: homeostatic model assessment for insulin resistance
IDF: International Diabetes Federation
LDL-C: low-density lipoprotein cholesterol
MetS: metabolic syndrome
METS-IR: metabolic score for insulin resistance
ML: machine learning
NPV: negative predictive value
PPV: positive predictive value
pre-MetS: pre-metabolic syndrome
SBP: systolic blood pressure
T2DM: type 2 diabetes mellitus
TG: triglycerides
TyG: triglyceride-glucose index
TyG-BMI: triglyceride-glucose body mass index
TyG-WC: triglyceride-glucose waist circumference
TyG-WHtR: triglyceride-glucose waist-to-height ratio
WC: waist circumference
WHtR: waist-to-height ratio
XGBoost: extreme gradient boosting
