# Supplementary material for: Classification of pre-metabolic syndrome and metabolic syndrome using machine learning and routine clinical variables in Thai adults
Source: PLoS One. 2026 Sep 28;21(9):e0359283. doi: 10.1371/journal.pone.0359283 (PMC13618934; doi:10.1371/journal.pone.0359283)
Supplement: Supplementary file 1 [file pone.0359283.s001.pdf]

S1 Fig. Study Population and Data Preparation.

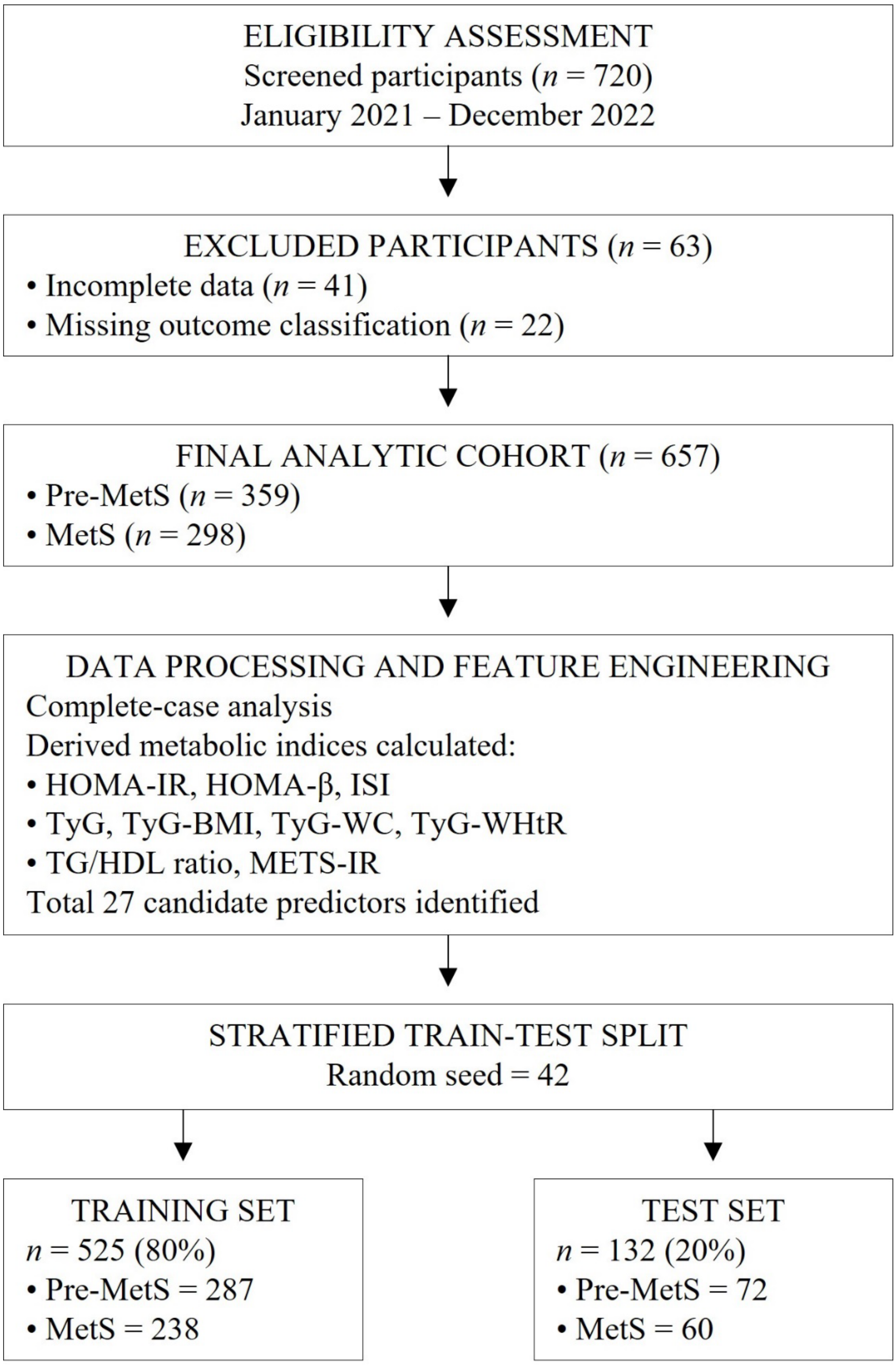

The workflow summarizes participant selection, outcome classification, feature engineering, train-test splitting, data standardization, and feature selection procedures performed before machine-learning model development. Participants were classified as pre-metabolic syndrome (pre-MetS) or metabolic syndrome (MetS) according to the International Diabetes Federation (IDF) criteria for Asian populations. Derived metabolic indices were calculated from routinely collected clinical measurements. The dataset was divided into training (80%) and testing (20%) subsets using stratified random sampling. Z-score normalization and

Boruta feature selection were performed using the training dataset only, and the resulting parameters were applied to the testing dataset to minimize information leakage.

Abbreviations: HOMA-IR, homeostatic model assessment of insulin resistance; HOMA-β, homeostatic model assessment of β-cell function; IDF, International Diabetes Federation; LDL-C, low-density lipoprotein cholesterol; MetS, metabolic syndrome; METS-IR, metabolic score for insulin resistance; pre-MetS, pre-metabolic syndrome; TG, triglycerides; TyG, triglyceride-glucose index; TC, total cholesterol; WC, waist circumference; WHtR, waist-to-height ratio.
