# Supplementary material for: Classification of pre-metabolic syndrome and metabolic syndrome using machine learning and routine clinical variables in Thai adults
Source: PLoS One. 2026 Sep 28;21(9):e0359283. doi: 10.1371/journal.pone.0359283 (PMC13618934; doi:10.1371/journal.pone.0359283)
Supplement: Supplementary file 2 [file pone.0359283.s002.pdf]

S2 Fig. Classification algorithm used to assign participants to pre-metabolic syndrome (pre-MetS) and metabolic syndrome (MetS) groups.

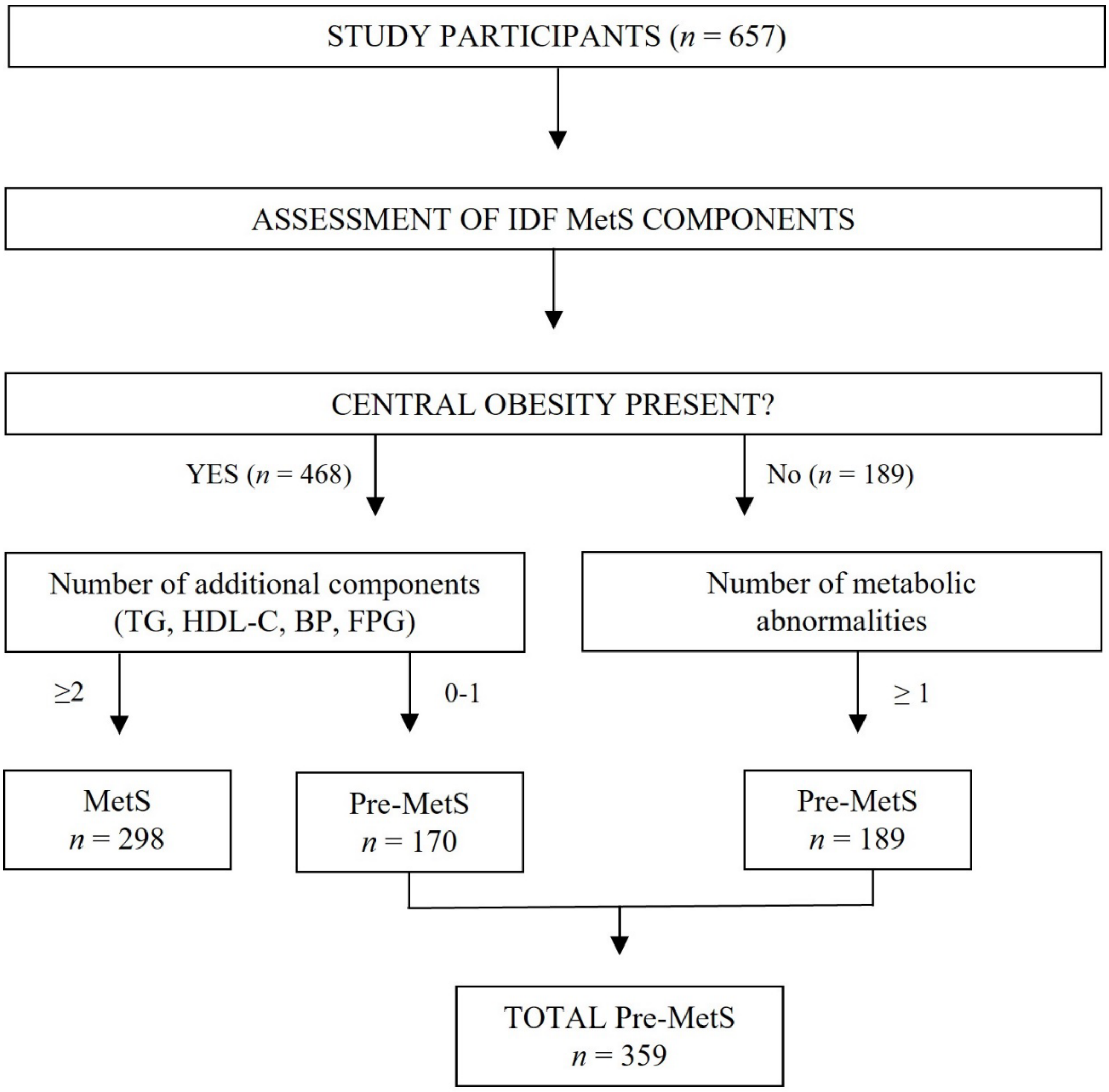

Classification algorithm used to assign participants to pre-metabolic syndrome (pre-MetS) and metabolic syndrome (MetS) groups. Participants were classified according to the International Diabetes Federation (IDF) criteria for metabolic syndrome. MetS was defined as the presence of central obesity together with at least two additional metabolic abnormalities, including elevated triglycerides, reduced high-density lipoprotein cholesterol (HDL-C), elevated blood pressure, or elevated fasting plasma glucose (FPG). Participants who did not meet the full IDF definition of MetS but had one or two metabolic abnormalities were operationally classified as having pre-MetS. Individuals with no metabolic abnormalities were outside the study scope and were not included in the analysis.

Abbreviations: BP, blood pressure; FPG, fasting plasma glucose; HDL-C, high-density lipoprotein cholesterol; IDF, International Diabetes Federation; MetS, metabolic syndrome; pre-MetS, pre-metabolic syndrome; TG, triglycerides.
