# Supplementary material for: Classification of pre-metabolic syndrome and metabolic syndrome using machine learning and routine clinical variables in Thai adults
Source: PLoS One. 2026 Sep 28;21(9):e0359283. doi: 10.1371/journal.pone.0359283 (PMC13618934; doi:10.1371/journal.pone.0359283)

S3 Fig. Machine Learning Model Development and Evaluation.

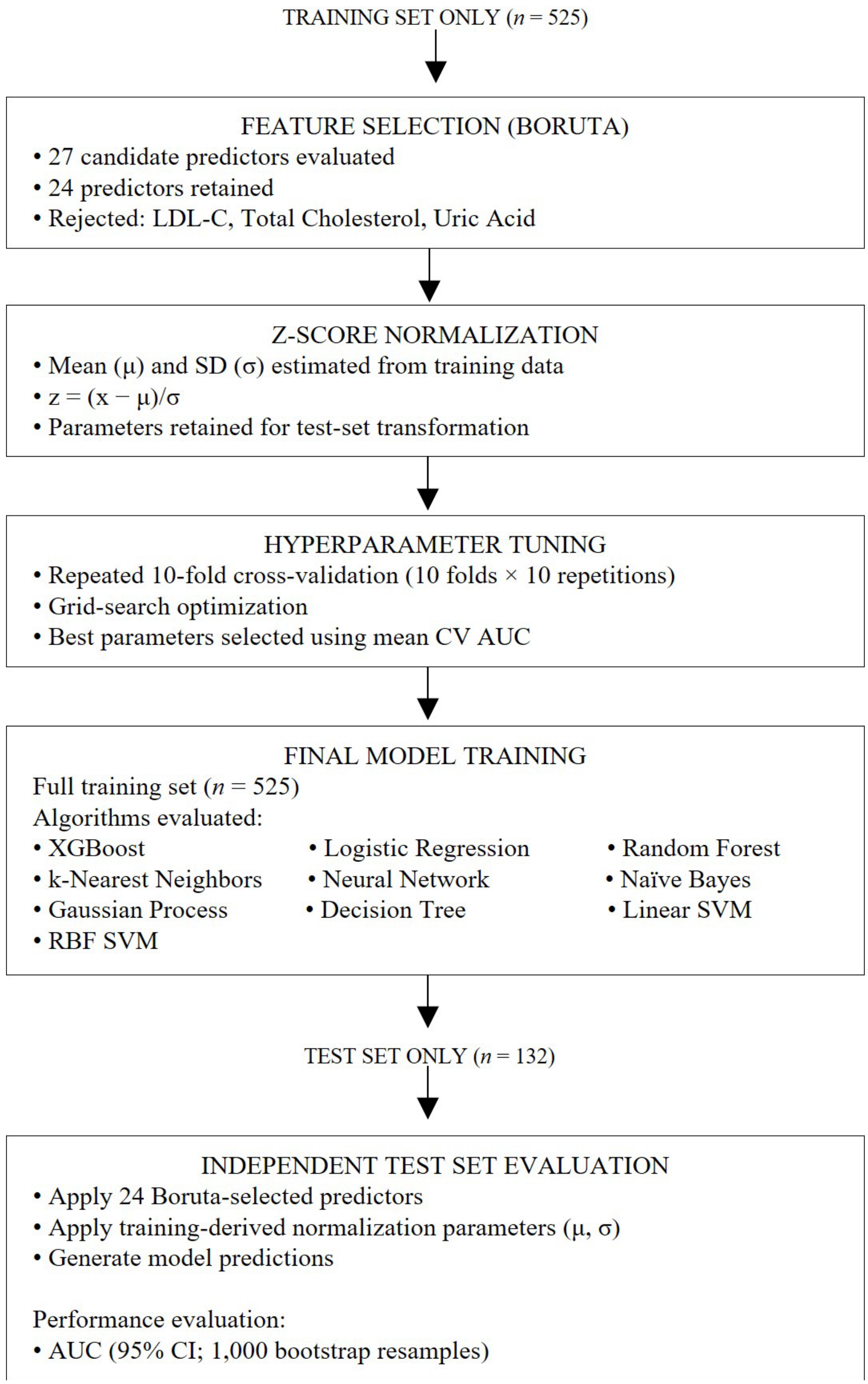

Supplement: Supplementary file 3 [file pone.0359283.s003.pdf]
