# Supplementary material for: Classification of pre-metabolic syndrome and metabolic syndrome using machine learning and routine clinical variables in Thai adults
Source: PLoS One. 2026 Sep 28;21(9):e0359283. doi: 10.1371/journal.pone.0359283 (PMC13618934; doi:10.1371/journal.pone.0359283)
Supplement: Supplementary file 4 [file pone.0359283.s004.pdf]

S4 Fig. Boruta feature selection: median importance scores and variable classification among candidate features.

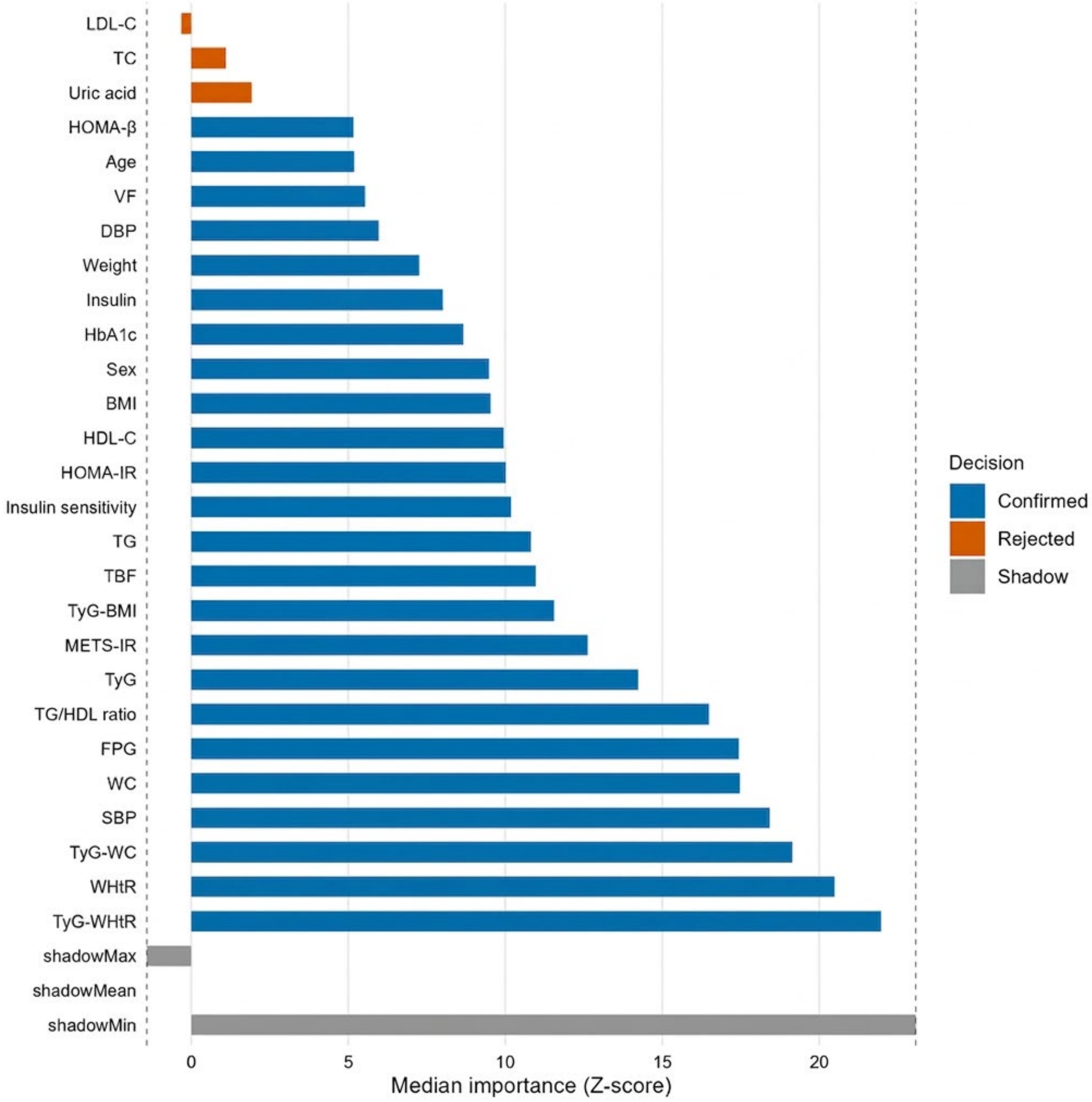

The horizontal bar plot shows the median importance (Z-score) of candidate predictor variables obtained using the Boruta feature-selection algorithm. Variables are ranked on the y-axis according to their median importance scores, with higher Z-scores on the x-axis indicating greater importance.

Variables are classified according to the Boruta decision rule and color-coded as confirmed (blue), tentative (yellow), rejected (orange), or shadow features (gray). Shadow features (shadowMin, shadowMean, and shadowMax) are randomly permuted copies of the original variables used as reference features for assessing variable importance. Variables with importance scores consistently exceeding those of the shadow features are classified as confirmed, whereas variables with importance scores comparable to or lower than those of the shadow features are classified as tentative or rejected.

Abbreviations: BMI, body mass index; DBP, diastolic blood pressure; FPG, fasting plasma glucose; HbA1c, glycated hemoglobin; HDL-C, high-density lipoprotein cholesterol; HOMA-β, homeostasis model assessment of β-cell function; HOMA-IR, homeostasis model assessment of insulin resistance; LDL-C, low-density lipoprotein cholesterol; METS-IR, metabolic score for insulin resistance; SBP, systolic blood pressure; TBF, total body fat; TC, total cholesterol; TG/HDL-C ratio, triglyceride-to-high-density lipoprotein cholesterol ratio; TyG, triglyceride-glucose index; TyG-BMI, triglyceride-glucose body mass index; TyG-WC, triglyceride-glucose waist circumference index; TyG-WHtR, triglyceride-glucose waist-to-height ratio index; VF, visceral fat; WC, waist circumference; WHtR, waist-to-height ratio.
