# Supplementary material for: Classification of pre-metabolic syndrome and metabolic syndrome using machine learning and routine clinical variables in Thai adults
Source: PLoS One. 2026 Sep 28;21(9):e0359283. doi: 10.1371/journal.pone.0359283 (PMC13618934; doi:10.1371/journal.pone.0359283)
Supplement: Supplementary file 5 [file pone.0359283.s005.pdf]

S5 Fig. Receiver operating characteristic (ROC) curves of machine learning models for classification of pre-metabolic syndrome (pre-MetS) and metabolic syndrome (MetS).

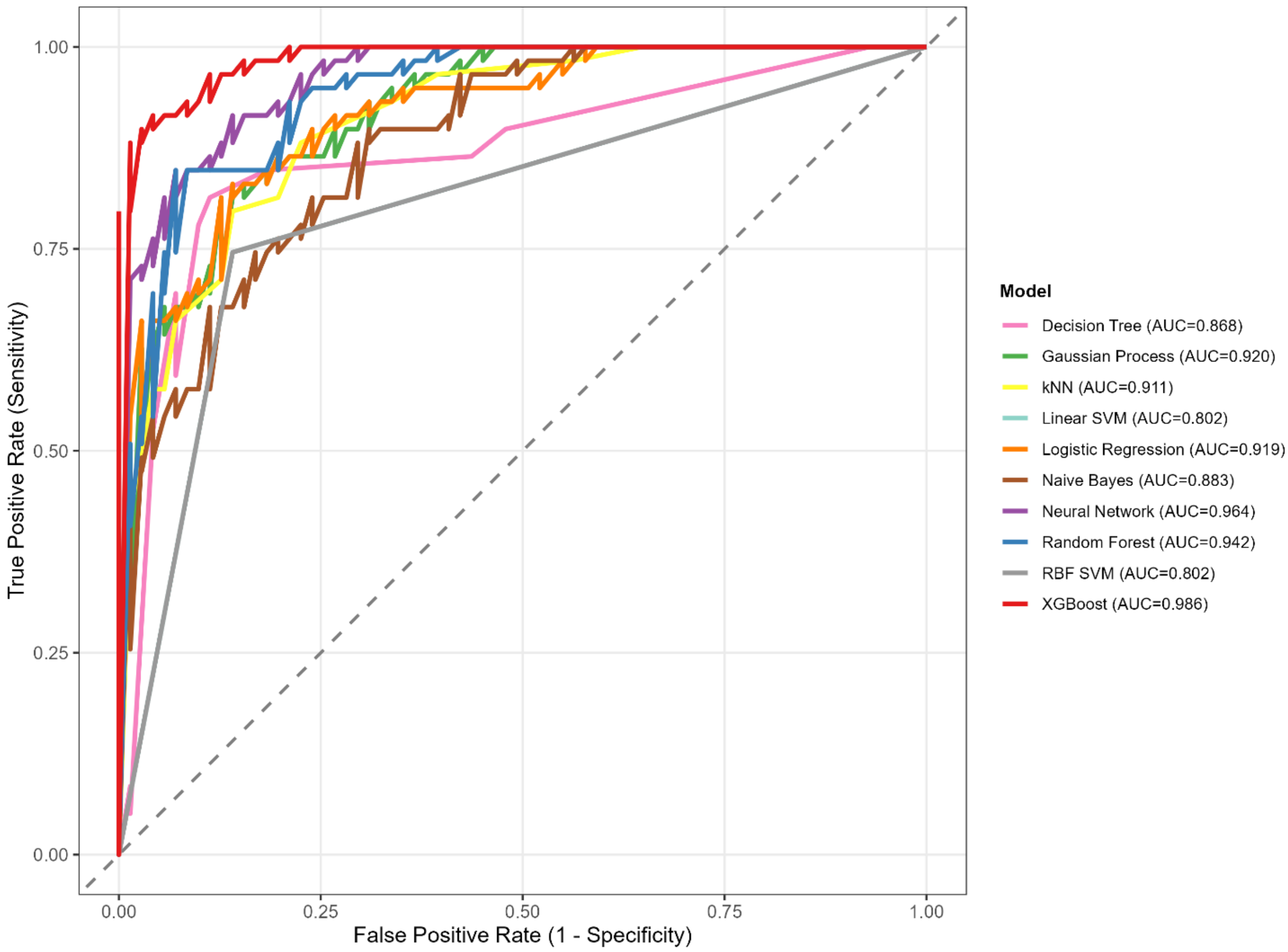

ROC curves illustrate the relationship between sensitivity (true positive rate) and 1 − specificity (false positive rate) for extreme gradient boosting (XGBoost), random forest, Gaussian process, neural network, logistic regression, k-nearest neighbors (kNN), decision tree, naïve Bayes, linear support vector machine (SVM), and radial basis function (RBF) SVM. The area under the ROC curve (AUC) for each model is shown in the legend. The diagonal dashed line represents no discriminatory ability (AUC = 0.5).

Abbreviations: AUC, area under the ROC curve; kNN, k-nearest neighbors; MetS, metabolic syndrome; RBF, radial basis function; SVM, support vector machine; XGBoost, extreme gradient boosting.
