# Supplementary material for: Classification of pre-metabolic syndrome and metabolic syndrome using machine learning and routine clinical variables in Thai adults
Source: PLoS One. 2026 Sep 28;21(9):e0359283. doi: 10.1371/journal.pone.0359283 (PMC13618934; doi:10.1371/journal.pone.0359283)
Supplement: Supplementary file 6 [file pone.0359283.s006.pdf]

S1 Table. Comparison of baseline characteristics between included and excluded participants.

**Characteristic**	**Included Participants (*n* = 657)**	**Excluded Participants (*n* = 63)**	***p*-value***
Age (years)	61.0 (52.0-68.0)	59.5 (50.0-67.0)	0.32
Sex (Male/Female)	237/420	22/41	0.87
BMI (kg/m²)	26.8 (23.5-30.5)	26.5 (23.0-30.2)	0.45
Waist circumference (cm)	88.0 (82.0-96.0)	87.5 (81.0-95.5)	0.38
SBP (mmHg)	128.0 (118.0-138.0)	126.5 (117.0-137.0)	0.41
DBP (mmHg)	78.0 (69.0-85.0)	77.0 (68.0-84.5)	0.52
FPG (mg/dL)	115.0 (95.0-135.0)	113.0 (94.0-134.0)	0.36
TG (mg/dL)	120.0 (85.0-165.0)	118.0 (84.0-163.0)	0.44
HDL-C (mg/dL)	49.0 (41.0-60.0)	50.0 (42.0-59.0)	0.56

* Continuous variables were compared using the Mann-Whitney U test, and categorical variables were compared using the chi-square test.

Abbreviations: BMI, body mass index; DBP, diastolic blood pressure; FPG, fasting plasma glucose; HDL-C, high-density lipoprotein cholesterol; IQR, interquartile range; SBP, systolic blood pressure; TG, triglycerides.

Note: No statistically significant differences were observed between included and excluded participants (all $P > 0.05$), suggesting that exclusion because of incomplete data was unlikely to introduce substantial selection bias.
