# Supplementary material for: Classification of pre-metabolic syndrome and metabolic syndrome using machine learning and routine clinical variables in Thai adults
Source: PLoS One. 2026 Sep 28;21(9):e0359283. doi: 10.1371/journal.pone.0359283 (PMC13618934; doi:10.1371/journal.pone.0359283)
Supplement: Supplementary file 7 [file pone.0359283.s007.pdf]

S2 Table. Frequency of individual metabolic syndrome diagnostic components in pre-MetS and MetS.

**MetS component (criterion*)**	**pre-MetS (*n* = 359)**	**MetS (*n* = 298)**	***p*-value†**
**Central obesity**			
Waist circumference ≥ cutoff, n (%)	170 (47.4%)	298 (100.0%)	<0.001
WHtR > 0.5, n (%)‡	251 (69.9%)	287 (96.3%)	<0.001
**Elevated triglycerides**	73 (20.3%)	174 (58.4%)	<0.001
**Reduced HDL-C**	63 (17.5%)	169 (56.7%)	<0.001
**Elevated blood pressure**	132 (36.8%)	234 (78.5%)	<0.001
**Elevated fasting plasma glucose**	182 (50.7%)	268 (89.9%)	<0.001

* MetS components were defined according to the International Diabetes Federation (IDF) criteria for Asian populations. Central obesity was defined as waist circumference ≥90 cm in men or ≥80 cm in women. Elevated triglycerides (TG) were defined as TG ≥150 mg/dL. Reduced high-density lipoprotein cholesterol (HDL-C) was defined as HDL-C <40 mg/dL in men or <50 mg/dL in women. Elevated blood pressure was defined as systolic blood pressure ≥130 mmHg and/or diastolic blood pressure ≥85 mmHg. Elevated fasting plasma glucose (FPG) was defined as FPG ≥100 mg/dL or previously diagnosed type 2 diabetes mellitus (T2DM). Because detailed medication records were unavailable, treatment-based components of the full IDF definition could not be directly applied.

† Comparisons between groups were performed using the **Chi-square test**. Because these variables constitute the diagnostic components used to classify participants into the pre-MetS and MetS groups, p-values are presented for descriptive purposes only and should not be interpreted as evidence of independent associations.

‡ WHtR > 0.5 is not part of the IDF diagnostic criteria for MetS and is presented as a descriptive measure of central adiposity.

Note: Pre-MetS was operationally defined as the presence of one or two metabolic abnormalities, regardless of whether central obesity was present, among participants who did not fulfill the complete IDF criteria for MetS. Thus, central obesity was not required for classification as pre-MetS. The pre-MetS group therefore included participants with central obesity and one additional metabolic abnormality as well as participants without central obesity who had one or two metabolic abnormalities.

Abbreviations: HDL-C, high-density lipoprotein cholesterol; IDF, International Diabetes Federation; MetS, metabolic syndrome; pre-MetS, pre-metabolic syndrome; FPG, fasting plasma glucose; SBP, systolic blood pressure; DBP, diastolic blood pressure; TG, triglycerides; T2DM, type 2 diabetes mellitus; WHtR, waist-to-height ratio.
