# Supplementary material for: Classification of pre-metabolic syndrome and metabolic syndrome using machine learning and routine clinical variables in Thai adults
Source: PLoS One. 2026 Sep 28;21(9):e0359283. doi: 10.1371/journal.pone.0359283 (PMC13618934; doi:10.1371/journal.pone.0359283)
Supplement: Supplementary file 8 [file pone.0359283.s008.pdf]

S3 Table. Hyperparameter search grids and final selected parameters for machine-learning models.

Model	Hyperparameter	Search Grid	Selected Value
Random Forest	mtry	2, 4, 6, 8, 10	10
	ntree	500 (fixed)	500
XGBoost	nrounds	50, 100, 150	100
	max_depth	3, 6, 9	6
	eta	0.05, 0.1, 0.2	0.1
	gamma	0 (fixed)	0
	colsample_bytree	0.8 (fixed)	0.8
	min_child_weight	1 (fixed)	1
	subsample	0.8 (fixed)	0.8
RBF SVM	sigma	0.01, 0.05, 0.1, 0.5	0.01
	C	0.5, 1, 2, 5	5
Linear SVM	C	0.1, 0.5, 1, 2, 5, 10	10
Logistic Regression	alpha	0, 0.25, 0.5, 0.75, 1	0.75
	lambda	0.0001, 0.00036, 0.00129, 0.00464, 0.0167, 0.0599, 0.215, 0.774, 2.78, 10	0.0001
k-Nearest Neighbors	k	3, 5, 7, 9, 11, 13, 15	13
Decision Tree	cp	0.001, 0.005, 0.01, 0.02, 0.05, 0.1	0.005
Neural Network	size	3, 5, 7, 10	3
	decay	0.001, 0.01, 0.1	0.1
Naïve Bayes	laplace	0, 0.5, 1	0
	usekernel	TRUE, FALSE	TRUE
Gaussian Process	sigma	0.01, 0.05, 0.1, 0.5, 1.0	0.05

Note: Hyperparameters were optimized using grid search with repeated 10-fold cross-validation (10 repetitions) on the training dataset. The search grids and final selected values are shown. Hyperparameters not included in the search grid were held constant at their default or prespecified values during model training.

Abbreviations: kNN, k-nearest neighbors; RBF, radial basis function; SVM, support vector machine; XGBoost, extreme gradient boosting; *mtry*, number of variables randomly sampled as candidates at each split; *ntree*, number of trees

.
