# Supplementary material for: Classification of pre-metabolic syndrome and metabolic syndrome using machine learning and routine clinical variables in Thai adults
Source: PLoS One. 2026 Sep 28;21(9):e0359283. doi: 10.1371/journal.pone.0359283 (PMC13618934; doi:10.1371/journal.pone.0359283)
Supplement: Supplementary file 9 [file pone.0359283.s009.pdf]

S4 Table. Baseline characteristics of participants in the training and test datasets.

Parameter	Training Set (n = 525)	Test Set (n = 132)	p-value*	Standardized Difference
**Demographics**				
Sex (Male/Female)	189/336	49/83	0.78	0.03
Age (years)	61.0 (52.0-68.0)	60.0 (51.0-68.0)	0.45	0.06
Anthropometrics				
Weight (kg)	68.0 (60.0-78.0)	67.0 (59.0-77.0)	0.42	0.06
BMI (kg/m²)	26.8 (23.5-30.5)	26.7 (23.4-30.4)	0.52	0.05
Waist circumference (cm)	88.0 (82.0-96.0)	88.5 (82.0-96.0)	0.38	0.07
WHtR	0.55 (0.52-0.59)	0.55 (0.52-0.59)	0.41	0.06
Total body fat (%)	33.0 (27.5-38.0)	33.5 (27.8-38.2)	0.44	0.06
Visceral fat	12.0 (7.0-17.0)	12.0 (7.0-17.0)	0.56	0.04
**Hemodynamics**				
SBP (mmHg)	128.0 (118.0-138.0)	127.0 (117.0-138.0)	0.42	0.06
DBP (mmHg)	78.0 (69.0-85.0)	78.0 (69.0-85.0)	0.56	0.04
Lipid profile				
TG (mg/dL)	120.0 (85.0-165.0)	119.0 (84.0-164.0)	0.41	0.06
HDL-C (mg/dL)	49.0 (41.0-60.0)	49.0 (41.0-60.0)	0.48	0.05
**Biochemical variables**				
FPG (mg/dL)	115.0 (95.0-135.0)	114.0 (94.0-134.0)	0.33	0.08
HbA1c (%)	5.9 (5.5-6.4)	5.9 (5.5-6.4)	0.48	0.05
Insulin (µIU/mL)	13.0 (9.0-20.0)	13.0 (9.0-20.0)	0.61	0.04
**Insulin resistance and sensitivity indices**				
HOMA-IR	3.5 (2.4-5.6)	3.5 (2.4-5.6)	0.52	0.05
HOMA-β	114.0 (72.0-176.0)	113.0 (71.0-175.0)	0.44	0.06
Insulin sensitivity	0.31 (0.29-0.34)	0.31 (0.29-0.34)	0.47	0.05
**Composite metabolic indices**				
TyG index	8.8 (8.5-9.2)	8.8 (8.5-9.2)	0.39	0.07
TyG-WC	800.0 (710.0-890.0)	805.0 (712.0-892.0)	0.44	0.06
TyG-BMI	262.0 (229.0-292.0)	265.0 (232.0-295.0)	0.40	0.06
TyG-WHtR	5.0 (4.6-5.5)	5.0 (4.6-5.5)	0.47	0.05
TG/HDL-C ratio	2.5 (1.5-3.8)	2.4 (1.5-3.7)	0.49	0.04
METS-IR	40.0 (35.0-47.0)	40.0 (35.0-47.0)	0.43	0.06
**Metabolic syndrome status**				
Pre-MetS, n (%)	287 (54.7%)	72 (54.5%)	0.87	0.02
MetS, n (%)	238 (45.3%)	60 (45.5%)	0.87	0.02

Data are presented as median (interquartile range [IQR]) for continuous variables and frequency (percentage) for categorical variables.

* Continuous variables were compared using the Mann-Whitney U test, and categorical variables were compared using the chi-square test.

Abbreviations: BMI, body mass index; DBP, diastolic blood pressure; FPG, fasting plasma glucose; HbA1c, glycated hemoglobin; HDL-C, high-density lipoprotein cholesterol; HOMA-IR, homeostasis model assessment of insulin resistance; HOMA-β, homeostasis model assessment of β-cell function; METS-IR, metabolic score for insulin resistance; MetS, metabolic syndrome; Pre-MetS, pre-metabolic syndrome; SBP, systolic blood pressure; TG, triglycerides; TyG, triglyceride-glucose index; TyG-BMI, triglyceride-glucose-body mass index; TyG-WC, triglyceride-glucose-waist circumference index; TyG-WHtR, triglyceride-glucose-waist-to-height ratio; WHtR, waist-to-height ratio.

Note: Standardized differences were used to assess balance between the training and test datasets. Values <0.10 indicate negligible imbalance, values of 0.10-0.20 indicate small imbalance, and values >0.20 indicate meaningful imbalance. All standardized differences were <0.10, indicating excellent balance between the training and test datasets following stratified random sampling. No statistically significant differences were observed between the training and test datasets for demographic, anthropometric, hemodynamic, biochemical, metabolic, or metabolic syndrome status variables (all $p > 0.05$).
