# Supplementary material for: Classification of pre-metabolic syndrome and metabolic syndrome using machine learning and routine clinical variables in Thai adults
Source: PLoS One. 2026 Sep 28;21(9):e0359283. doi: 10.1371/journal.pone.0359283 (PMC13618934; doi:10.1371/journal.pone.0359283)
Supplement: Supplementary file 10 [file pone.0359283.s010.pdf]

S5 Table. Sensitivity analyses of XGBoost performance after exclusion of overlapping and MetS-related features

Analysis	Excluded Features	Retained Features (*n*)	AUC	Accuracy	Sensitivity	Specificity	PPV	NPV	F1-score	Brier Score
Primary analysis	None	24	0.986 (0.970-0.997)	0.932 (0.885-0.969)	0.882 (0.792-0.958)	0.973 (0.930-1.000)	0.965 (0.906-1.000)	0.909 (0.835-0.964)	0.921 (0.864-0.967)	0.050 (0.026-0.080)
Sensitivity analysis 1	TyG-WC, TyG-WHtR, and METS-IR	21	0.912 (0.878–0.946)	0.847 (0.801–0.885)	0.763 (0.710–0.810)	0.915 (0.877–0.944)	0.889 (0.847–0.922)	0.814 (0.764–0.857)	0.821 (0.774–0.862)	0.094 (0.064–0.134)
Sensitivity analysis 2†	All MetS diagnostic components and related derived indices†	12	0.875 (0.836–0.914)	0.792 (0.742–0.835)	0.718 (0.663–0.768)	0.856 (0.812–0.893)	0.831 (0.779–0.875)	0.763 (0.716–0.806)	0.771 (0.720–0.817)	0.142 (0.106–0.184)
Sensitivity analysis 3‡	All MetS diagnostic components, related derived indices, and WHtR‡	11	0.861 (0.819–0.903)	0.776 (0.724–0.822)	0.695 (0.639–0.747)	0.843 (0.798–0.882)	0.817 (0.764–0.863)	0.742 (0.694–0.786)	0.752 (0.699–0.801)	0.158 (0.120–0.202)

Notes: Values are presented as performance estimates with 95% confidence intervals (95% CIs). Hyperparameter tuning was performed using repeated 10-fold cross-validation with ten repetitions within the training dataset. Final model performance was evaluated in the independent hold-out test dataset. Ninety-five percent confidence intervals for all performance metrics were estimated using 1,000 bootstrap resamples. MetS was designated as the positive class. Models are ranked according to AUC. A probability threshold of 0.50 was used for binary classification. Lower Brier scores indicate better calibration.

† **Sensitivity analysis 2** excluded all variables directly used in the International Diabetes Federation (IDF) definition of metabolic syndrome (MetS), including waist circumference (WC), systolic blood pressure (SBP), diastolic blood pressure (DBP), fasting plasma glucose (FPG), triglycerides (TG), and high-density lipoprotein cholesterol (HDL-C), together with derived indices incorporating these components (TG/HDL-C ratio, TyG, TyG-BMI, TyG-WC, TyG-WHtR, and METS-IR).

‡ **Sensitivity analysis 3** additionally excluded waist-to-height ratio (WHtR), which incorporates waist circumference, to evaluate whether classification performance remained after removal of all MetS diagnostic components and related anthropometric derivatives.

Abbreviations: AUC, area under the receiver operating characteristic curve; BMI, body mass index; CI, confidence interval; DBP, diastolic blood pressure; FPG, fasting plasma glucose; HDL-C, high-density lipoprotein cholesterol; HOMA-IR, homeostasis model assessment of insulin resistance; HOMA-β, homeostasis model assessment of β-cell function; METS-IR, metabolic score for insulin resistance; MetS, metabolic syndrome; NPV, negative predictive value; PPV, positive predictive value; SBP, systolic blood pressure; TG, triglycerides; TyG, triglyceride-glucose index; TyG-WC, triglyceride-glucose waist circumference index; TyG-WHtR, triglyceride-glucose waist-to-height ratio; WC, waist circumference; WHtR, waist-to-height ratio; XGBoost, extreme gradient boosting.
